# Color and morphological differentiation in the Sinaloa Wren (*Thryophilus sinaloa*) in the tropical dry forests of Mexico: The role of environment and geographic isolation

**DOI:** 10.1371/journal.pone.0269860

**Published:** 2022-06-23

**Authors:** Andreia Malpica, Luis Mendoza-Cuenca, Clementina González

**Affiliations:** 1 Instituto de Investigaciones sobre los Recursos Naturales, Universidad Michoacana de San Nicolás de Hidalgo, Michoacán, México; 2 Laboratorio de Ecología de la Conducta, Facultad de Biología, Universidad Michoacana de San Nicolás de Hidalgo, Michoacán, México; University of Akron, UNITED STATES

## Abstract

The role and the degree to which environment and geographic isolation contribute to phenotypic diversity has been widely debated. Here, we studied phenotypic variation (morphology and plumage reflectance) in the Sinaloa Wren, an endemic bird distributed throughout the tropical dry forest (TDF) on the Mexican pacific slope where a pronounced variability in environmental conditions has been reported. In particular, we aimed: 1) to characterize phenotypic variation between subspecies; 2) to analyze the relationship between phenotypic and environmental variation in the context of classic ecogeographic rules, such as Bergmann’s, Allen’s, Gloger’s, and Bogert’s, and to quantify the relative roles of environment and geographic isolation and their interaction in shaping phenotypic variation; and 3) to test for niche conservatism between subspecies. Our data revealed significant differences among subspecies morphology and plumage reflectance. The environment explained a higher proportion of the morphological variation, while geography explained a smaller proportion. However, variation in plumage reflectance was mainly explained by the joint effect of geography and environment. Our data did not support for Bergmann´s and Allen´s rule. However, longer tails and wings were positively associated with higher elevations, larger tarsus and culmens were positively related to higher latitudes and to greater tree cover, respectively. Our data partially supported Gloger´s rule, where darker plumages were associated with more humid environments. The effects of temperature on plumage coloration were more consistent with Bogert´s rule. In addition, we found darker plumages related to higher levels of UV-B radiation. Finally, niche divergence was detected between *T*. *s*. *cinereus* and *T*. *s*. *sinaloa* vs. *T*. *s*. *russeus*. In a continuously distributed ecosystem such as the TDF on the pacific slope of Mexico, the environmental conditions and geographic isolation have played an important role in promoting phenotypic differentiation in the Sinaloa Wren.

## Introduction

In birds, phenotypic traits such as plumage coloration, morphological traits (e.g. body size), songs and behavioral displays are involved in conspecific recognition, as well as mate choice [1]. Divergence of these traits could therefore be an important driver of premating reproductive isolation [2,3]. Phenotypic divergence between populations might occur through local adaptation, as a result of natural selection when populations evolve advantageous traits under their local environment, or by phenotypic plasticity, where environmental variation influences phenotype by creating non-heritable trait variation [4]. Moreover, phenotypic divergence might also result from neutral effects of genetic drift [5], where the amount of gene flow between populations decreases as the geographic distance between them increases (e.g. [6]). The effects of these mechanisms are not mutually exclusive, since both could influence phenotypic differentiation between populations through the evolutionary history of species [7]. Studies that quantify the proportion of phenotypic variation explained by geography and environment are therefore essential in order to understand the factors that drive morphological variation (e.g. [8,9]), which can eventually contribute to speciation.

Geographic patterns of morphological and color plumage variation in birds have been associated with climate, latitude, elevation and resource availability [10–13]. For example, according to Bergmann’s rule, temperature has been suggested as the principal factor shaping latitudinal body size variation, where larger bodies are expected in colder climates as a thermoregulatory response to prevent heat dissipation [14]. Likewise, Allen’s rule predicts that homeothermic animals should have shorter appendages (e.g. tarsus and culmen) in cooler climates in order to minimize heat loss [15]. However, other factors have also been suggested to influence phenotypic variation; for instance, longer wings and tails have been positively associated with higher elevations where low atmospheric pressures and strong air currents prevail [16–18], which helps to increase flight efficiency, as well as contributing to balance and lift [19,20]. Moreover, environmental variables such as precipitation could impact habitat characteristics such as understory density and overall forest interior structure [21], which can influence tarsus, wing and tail morphology since these traits are respectively related to foraging, flight performance and maneuverability [22–24]. In addition, longer bills have been positively related to deeper substrates in more humid habitats [25], and to warm and humid habitats as a mechanism for heat dissipation [26,27]. Finally, temperature and humidity also have an effect on melanin-based feather coloration (e.g. [28,29]). According to the simple version of Gloger’s rule, darker colorations are expected in more humid and warmer areas, and brighter colorations in cooler and dryer conditions [30,31] while, according to Bogert’s rule [32], darker colorations are expected in colder habitats in order that individuals can absorb more solar radiation, with lighter colorations expected in warmer habitats [33–35]. In addition, recent studies have demonstrated the implication of other factors such as ultraviolet radiation in explaining melanin production and variation that may be a source of oxidative stress which can affect overall plumage coloration [29].

**Fig 1:**
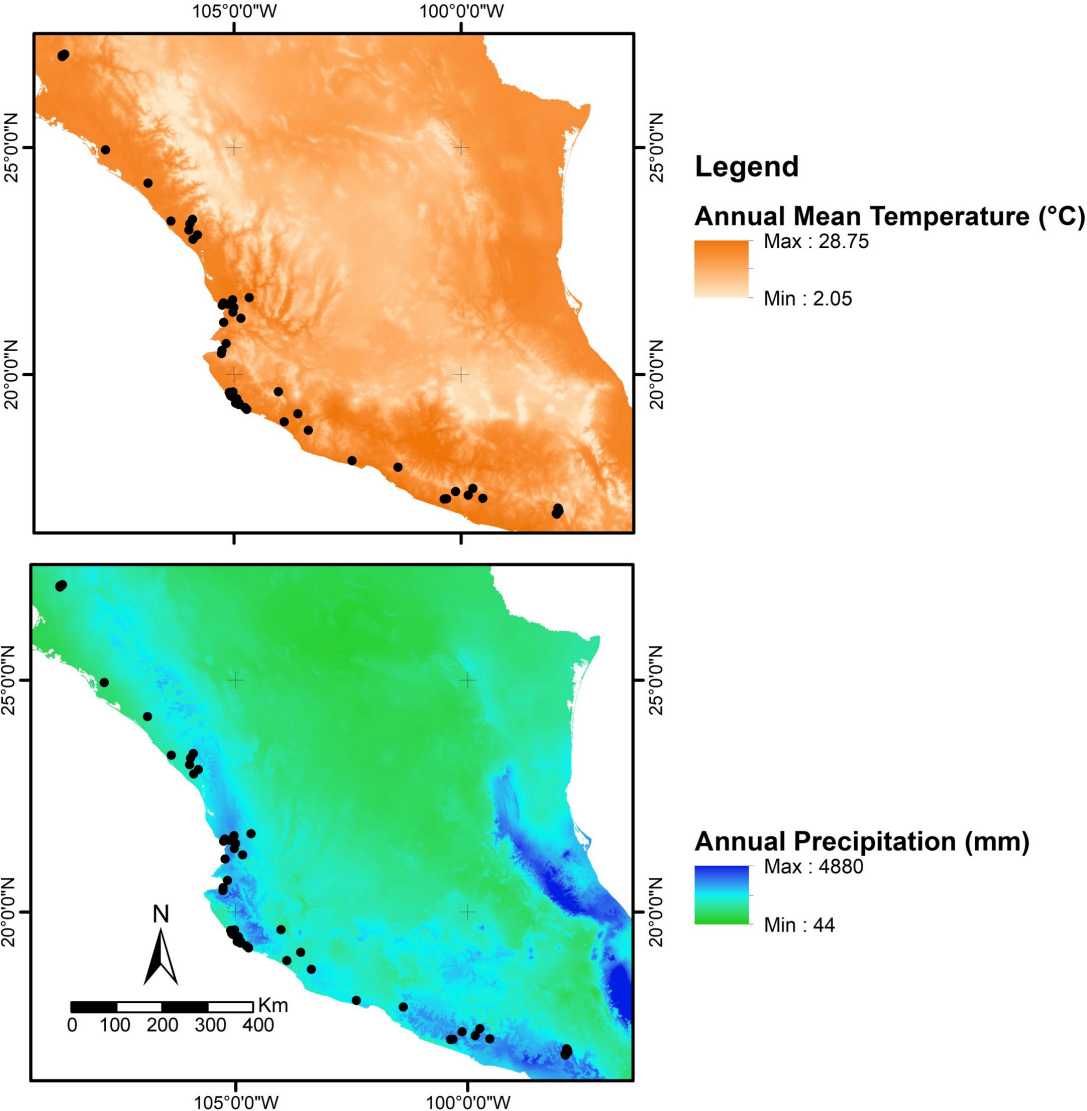
Environmental variation across the species distribution. BIOCLIM variables retrieved from WorldClim and projected into maps, showing (A) annual mean temperature (bio1) and (B) annual precipitation (bio12) across sampling localities of the Sinaloa Wren (black dots).

Tropical dry forest (TDF) is one of the most extensive ecosystems worldwide and is well represented in tropical and subtropical zones. In Mexico, TDF is almost continuously distributed over extensive areas along the Pacific slope from Sonora to Chiapas [36], with no apparent geographic barriers to dispersal (except for contemporary fragmentation) among populations of terrestrial vertebrates, including birds. The Mesoamerican TDFs are recognized as terrestrial ecosystems with high levels of species richness, endemism and specialization [37,38]. However, the factors that drive this huge diversity are poorly understood. Despite their recognition as a biodiversity hotspot, TDFs are highly threatened by human impact and the actions undertaken for their conservation have been minimal to date [39,40]. In Mexico, TDF is the second richest habitat for bird species, and the forests along the Pacific slope are home to several endemic bird species [41], most of which show subspecific variation (e.g. [42]). TDFs are characterized by marked seasonality, with a dry season from November to May in which most of the vegetation loses its leaves, and a rainy season from June to October where most of the rainfall occurs. Moreover, high variability in the environmental conditions have been reported (see description in methods) throughout the TDF distribution along the Mexican Pacific slope, including the temperature and precipitation regimes (Fig 1), as well as in the vegetation structure and composition [43–45]. This high variability could expose populations to different selective (environmental) pressures that might explain phenotypic divergence within some species.

Most studies of bird phenotypic variation have been conducted in the context of species distributed along obvious environmental gradients (e.g., [46–52]. However, the effects of environment and geographic isolation on the phenotypic variation of species that inhabit single and continuously distributed ecosystems along latitudinal gradients of environmental conditions have been poorly explored (but see [21,53]). Studies of phenotypic differentiation accompanied by niche conservatism (i.e. retention of ancestral environmental niche) and niche divergence tests (i.e. adaptation to different ecological conditions) among subspecies have been helpful to identify the processes that can lead to lineage differentiation [54]. Specifically, such multi-perspective evidence could help to identify the role of ecological speciation, in which divergent natural selection promotes diversification through adaptation to new environments [55].

The Sinaloa Wren (*Thryophilus sinaloa*, Aves: Troglodytidae) is an endemic bird from Mexico [56], closely associated with TDFs along the Pacific slope. To date, three subspecies have been described along the distribution of the species based on subtle differences in melanin-based plumage coloration [57–59]. These subspecies inhabit areas within the TDF that differ significantly in terms of environmental conditions (see description in methods), which could subject their populations to divergent selective pressures. In this study, we tested the ecogeographic Allen’s, Bergmann’s, Gloger’s and Bogert’s rules, as well as other suggested environmental factors, to explain phenotypic variation in the Sinaloa Wren along its distribution, and quantified the relative role of environment and geography in shaping phenotypic variation. Specifically, we: 1) characterized phenotypic variation (morphology and plumage reflectance) between sexes and among subspecies, 2) analyzed the relationship between phenotypic and environmental variation in the context of classic ecogeographic rules and quantified the relative roles of environment and geography, as well as their interaction, in shaping phenotypic variation, and 3) tested for niche conservatism among subspecies. According to Bergmann’s rule, we expected larger bodies (using wing chord as a proxy for body size) to be related to colder climates, while with Allen’s rule, we expected shorter tarsi and culmens in colder climates. Since other factors have been proposed to influence morphological differences, we expected longer wings and tails to prevail in more open habitats and at higher elevations. According to Gloger’s rule, we expected darker plumages to be related to humid and warmer conditions. In contrast, according to Bogert’s rule, we expected darker colors in colder climates, based on the thermoregulatory advantages that darker plumages provide by absorbing more solar radiation. We also expected darker plumage colorations in places with more UV-B radiation as a mechanism of UV protection. Finally, we expected niche divergence between subspecies as a potential adaptation to different ecological conditions. Our results will help to better understand the mechanisms that promote phenotypic variation of species that inhabit this biodiverse ecosystem that is home to many endemic birds, but is also one of the most threatened habitats worldwide as a result of human perturbation.

## Methods

### Study species

The Sinaloa Wren is an insectivorous medium-sized wren that mainly inhabits the understory of the TDF [56,60]. It is endemic to western Mexico, and its distribution spans from Sonora and south Chihuahua to western Oaxaca along the Pacific slope, and through the Río Balsas drainage system (S1 Fig, [61]), covering a linear distance of approximately 2,000 km. The sexes are similar, but the males are slightly larger and heavier than females [62]. Three subspecies are currently recognized in the literature (Figs 3B and S1): the nominate *Thryophilus sinaloa sinaloa* [57] occurs from central Sonora and western Durango south to Michoacán, *Thryophilus sinaloa cinereus* [58] is distributed in northwest Mexico, from northeastern Sonora and western Chihuahua to northern Sinaloa, and *Thryophilus sinaloa russeus* [59] occurs in Guerrero and western Oaxaca. These subspecies have been described based on subtle differences in melanin-based plumage coloration and color intensity. Phillips [63] suggested an undescribed subspecies in the coastal regions from western Nayarit to Colima or Michoacán, based on color differences, particularly on the flanks.

### Study sites

In Mexico, TDFs are developed between 0 and 1900 m asl, most frequently below 1500 m asl [45]. The most distinctive characteristic of these forests is their marked seasonality, presenting a severe dry season of five to eight consecutive months (generally between November and May), depending on the geographic location and a rainy season (generally between June to October) during which most of the rainfall occurs [45]. Annual mean temperature ranges from 23.1°C during the dry season to 26.4°C during the rainy season (Fig 1). Annual precipitation ranges from 12 mm/yr during the dry season to 758 mm/yr during the rainy season (Fig 1). One adaptation of the vegetation to this marked seasonality is the senescence of leaves during the dry season and production of new foliage during the rainy season [64], producing a dramatic change of appearance between these two seasons.

The northern extreme of the distribution of the Sinaloa Wren at the pacific Mexican coast, where the subspecies *cinereus* is distributed, shows a semiarid climate with the driest conditions ([65]; lower annual precipitation, 690 mm/yr; lower evapotranspiration during the dry season, 1.76 mm), lower UV-B radiation, 4,498 J/m^-2^/day^-1^ and intermediate temperature (23.8°C). In this area, the tree cover is 30%, and the trees lose almost all their leaves, which is reflected in low values of the Normalized Difference Vegetation Index (NDVI = 0.36, in a scale from 0 to 1, where 1 is greener) during the dry season, resembling an inhospitable habitat with “dead” vegetation where the understory has an intricate pattern due to the presence of climbing vines. The center of the distribution, where the subspecies *sinaloa* is distributed, presents intermediate conditions (1,084 mm/yr of precipitation, 16.80 mm of evapotranspiration during the dry season, 4,983 J/m^-2^/day of UV-B radiation), except for temperature, which presents the highest annual mean values (24.9°C). Although the trees also lose almost all their leaves in this area, values of NDVI during the dry season are also intermediate (0.58), as is the tree cover (44%). In contrast, the southern extreme of the distribution of the Sinaloa Wren, where the *russeus* subspecies is distributed, presents the most humid conditions ([65]; 1,500 mm/yr of precipitation, 24.73 mm of evapotranspiration during the dry season), the highest UV-B radiation (5,202 J/m^-2^/day), and intermediate temperature (23.73°C). The TDFs are denser in these areas, hosting many spiny plants [66] and showing a tree cover of 45%. The vegetation does not lose its entire foliage during the dry season, and presents the highest values of NDVI (0.67).

In contrast to the dry season, the TDFs are more homogeneous during the rainy season. Values of NDVI during the rainy season were higher and almost invariable across the distribution of the species (*cinereus* = 0.84, *sinaloa* = 0.82 and *russeus* = 0.82), reflecting the production of new foliage across the entire distribution of the TDFs. Mean evapotranspiration during the rainy season is also similar, at 45, 35 and 33 mm in the *cinereus*, *sinaloa* and *russeus* collecting sites, respectively.

### Field procedures and molecular sexing

We captured 147 individuals of *Thryophilus sinaloa* from 34 localities (S1 Table), covering most of the distributional range of the species. From each bird captured in mist-nets (12 x 2.5 m x 36 mm), we collected three drops of blood by venipuncture of the sub-brachial vein and stored these samples in Queen’s lysis buffer at -20°C in the laboratory for sex determination. We measured five morphological traits: 1) wing chord (from the carpal joint to the tip of the longest unflattened primary); 2) tail length (from the insertion of the two central rectrices to the tip of the longest rectrix); 3) total head length (from the occipital crest to the tip of the bill); 4) exposed culmen (from the feather base above the nostrils to the tip of the bill); and 5) tarsus length (from the inner bend of the tibiotarsal articulation to the base of the toes). Wing and tail measurements were taken with a wing ruler to the nearest 1 mm, while the rest of the measurements were taken with a dial caliper accurate to 0.1 mm. Finally, we collected five feathers from the back, flanks and head (crown) and the two outer rectrices for reflectance measurements. Since the back and flanks patches cover greater plumage areas, we took the interscapular region as a reference to collect back feathers and the lateral body apterium zone to collect flank feathers. Furthermore, we obtained morphological measurements (n = 35 males and 20 females) from ornithological collections: the Colección Nacional de Aves (IBUNAM), Colección Ornitológica (UMSNH) and Colección Regional de Aves (UAGro). We also obtained feather samples (n = 15) donated from IBUNAM and UAGro, and one outer rectrix collected from 5 individuals from Oaxaca.

Since there is no formal criterion with which to distinguish males and females in the Sinaloa Wren, we performed sex determination of the captured individuals using molecular techniques (polymerase chain reaction, PCR), following the method outlined in Çakmak et al. [67]. Briefly, the highly conserved chromodomain helicase DNA binding gene (CHD), located on the W chromosome and its homologous on the Z chromosome, is amplified using specific primers (P2/P8) [68]. This amplification results in distinct banding patterns on agarose gel as a result of length differences in intronic regions within this gene [67,68]. Given that male birds are homogametic (ZZ), whiles females birds are heterogametic (ZW), PCR products with double bands were scored as females, while PCR products with a single band were scored as males. Band patterns were confirmed with 23 tissue samples donated by the Burke Museum from individuals previously sexed by gonads description. DNA extraction was performed using the DNeasy Blood and Tissue kit (Qiagen®), following the manufacturer’s protocol, adding 80–100μL of blood, in a final elution volume of 50μL.

All sampling activities adhered to the guidelines on the use of wild birds in research formulated by the Ornithological Council, and had the permission of Mexico’s Secretaría de Medio Ambiente y Recursos Naturales, Subsecretaría de Gestión para la Protección Ambiental, Dirección General de Vida Silvestre (permit numbers: SGPA/DGVS/10390/17 and SGPA/DGVS/05374/19).

### Plumage reflectance analyses

To characterize variation in plumage reflectance between sexes and subspecies, a total of 124 adult specimens of *T*. *sinaloa* were included in the analyses. Reflectance measurements were taken from one feather collected from each of four plumage patches: crown, back, flanks and tail. Five feathers from each body patch were overlapped in the same direction and brushed to ensure that the barbules were in the same position with the exception of tail rectrices. Since the rectrices of the Sinaloa Wren have a brown and black banded pattern, reflectance measurements were taken from a single feather at the 3^rd^ or 4^th^ brown band. Each feather was measured at three different points covering most of the feather area. We used an RPH-1 reflection probe holder (Ocean Optics, Dunedin, FL) where the optic fiber was placed at 90°. For twenty-four specimens, we compared reflectance curve measurements taken from the collected feathers and directly from each body part of the specimens. Collected feathers preserved the original reflectance curve as found in the specimens. Reflectance measurements were taken with an Ocean Optics Jaz-El200 spectrophotometer (Ocean Optics), coupled with a Premium QR600-7-SR125F optic fiber, collecting values at wavelengths of between 300 and 700 nm for all feathers. Measurements were standardized to a WS-1 diffuse white standard (Ocean Optics). We coded raw data files of measured reflectance spectra by subspecies and processed these in the R package *pavo* ver. 2.6.1 [69]. The values of three spectra from each measured feather were averaged using the *aggspec* function. Electrical noise from reflectance spectra was removed using the opt = “smooth” (span = 0.35) argument. To correct negative values from the raw spectral data, we used the fixneg = “addmin” argument. Both of these processes used the *procspec* function.

Reflectance data were analyzed using three different approaches: 1) quantification of plumage coloration in the avian ultraviolet-visible wavelength range (hereafter, avian perspective; [70]), 2) quantification of plumage color in the human visible UV wavelength range (hereafter, colorimetric variables; [71]), and 3) measurement of plumage color discrimination in the avian visual space using a receptor noise model (hereafter, receptor noise model). To quantify coloration from the avian visual perspective, we plotted each plumage patch in the tetrahedral color space [70], which represents the possible avian color space based on relative stimulations of the four retinal cone types. Raw reflectance spectra were processed using the average ultraviolet sensitive avian system = “avg.uv”, and the “ideal” illuminant arguments of the *vismodel* function were used to determine the relative stimulation of the four avian cones and the calculation of quantum catches [70,72]. The cone stimulation values (u, s, m, l) were converted into a vector of three angles, which allows location of the points in the tetrahedral color space [70]. Three standard color variables were calculated from the avian tetrachromatic color space (avian perspective): hue theta (*h theta*), r vector (*r vec*), and maximum chroma (*r max*). Hue theta is the angle in radians that quantifies colors detected by the long-, medium-, and short-wavelength-sensitive cones [70]. Hue theta values ranged from–π to + π; in our data set, the values ranged approximately from 0 to –0.2, where values near –0.2 indicate red-shifted colors and values near 0 denote yellow-shifted colors [73]. The chroma or saturation of a color is given by the magnitude of *r*, which describes how a color differs from achromatic white/black [70]. The r vector (*r vec*) measures saturation or distance from the achromatic center, and maximum chroma (*r max*) measures the maximum r vector achievable for the hue of the color [70]. Higher values of *r vec* and *r max* indicate more saturated colors. We also obtained two colorimetric variables: mean brightness (*B2*: average reflectance over all wavelengths) and chroma (*S8*: relative difference between max and min reflectance considering the average brightness of the spectrum; [71]). Using the five color variables (hue theta, saturation, maximum chroma, brightness and chroma), we first compared individuals by sex within each subspecies and, after verifying that they did not differ statistically with a Wilcoxon-Mann-Whitney test (results not shown), we pooled the sexes together in subsequent analyses.

Finally, to assess whether birds could discriminate colors within plumage patches among subspecies, we measured plumage color discrimination in the avian space using a receptor noise model [74]. This model calculates the chromatic distance in the avian color space (Δ*S*) between the reflectance of two plumages. The Δ*S* represents a threshold of discrimination between two colors and is expressed in terms of JNDs (just noticeable differences). A theorical threshold of 1.0 JND represents the distance in the avian color space at which two colorful traits are quite chromatically distinct. Values < 1.0 JND indicate plumage patches that are probably indistinguishable, while values > 1.0 JND indicate plumage regions that are chromatically discernible between subspecies [74].

### Environmental data collection

We used 19 BIOCLIM variables from the WorldClim data base [75], which express mean, seasonal, and extreme conditions of temperature and precipitation. Climatic variables were obtained for each locality from which morphological and plumage reflectance data were obtained. Additionally, we included elevation data gathered from SRTM (Shuttle Radar Topography Mission, 90 m resolution; DOI number: /10.5066/F7PR7TFT), retrieved by *raster* package [76]. We also included three vegetation variables from the Terra Moderate Resolution Imaging Spectroradiometer (MODIS): percentage tree cover TREE (MOD44B product: [77]), total evapotranspiration ET (MOD16A2 product: [78]) and Normalized Difference Vegetation Index NDVI (MOD13Q1 product: [79]), downloaded with the aid of *MODISTools* R package [80]. Since TDFs are characterized by a marked seasonality, we chose data for NDVI and ET corresponding to the peak of the dry (April, hereafter, ET_dry and NDVI_dry) and rainy (August, hereafter, ET_rain and NDVI_rain) seasons, based on precipitation data reported in Trejo-Vázquez [65]. Finally, we obtained annual mean ultraviolet-B radiation (UV-B, wavelengths of 280–315 nm) from the gIUV database [81] for use in the analyses.

### Statistical analyses

#### Phenotypic variation

We performed Wilcoxon-Mann-Whitney tests [82] to document morphology and plumage coloration differences between sexes, using sex as fixed factor and each morphological trait measured, as well as each reflectance measure for each plumage patch, as dependent variables. To document morphology and plumage coloration differences between subspecies, we performed Kruskal-Wallis tests [83] using subspecies as fixed factors and morphology and reflectance data as dependent variables. Conover-Iman tests for multiple comparisons [84] were performed using the *conover*.*test* package. For both analyses, Bonferroni corrections were applied in order to correct for multiple simultaneous comparisons [85]. These analyses were performed in Rstudio ver. 1.2.1335 (R Studio Team 2016).

Color discrimination in the avian visual space (receptor noise model) was performed between pairs of subspecies for each of the four plumage patches separately. Following Maia and White [86], we estimated the geometric average of the Δ*S* values for all comparisons and calculated 95% confidence intervals (CI) performing a bootstrap using the function *bootcoldist* in *pavo*.

#### Relationship between phenotypic variation and environmental variables

To test the specific hypotheses about the relationship between environmental and phenotypic variation along the Sinaloa Wren distribution in the context of ecogeographic rules, as well as other factors that have been proposed to influence morphological variation (see Introduction), we performed a series of Generalized Linear Mixed-Effect Models (GLMM) with a Gaussian distribution and logit function. We constructed models including tree cover, elevation, latitude, ET_dry, ET_rain, annual mean temperature, annual precipitation and sex as fixed factors, with sampling site as the random effect, data collection method (field or museum) as a covariable, and wing chord (as a proxy of body size) for Bergmann’s, tarsus and culmen for Allen’s and tails (along with the other traits to test for alternative hypothesis) as response variables. To test for Gloger’s and Bogert’s rules of color variation, we constructed the model using locality-specific environmental variables related to temperature, precipitation, humidity (annual mean temperature, annual precipitation, ET_dry, ET_rain), greenness of vegetation (NDVI_dry, NDVI_rain) as fixed factors and mean brightness (*B2*) for each plumage patch as response variables. In order to account for the non-independence of individual measurements within a locality, we used sampling site as the random effect. Non-significant explanatory variables in the full model were backward eliminated to simplify the model. To test for the specific hypothesis of UV-B radiation in shaping patterns of melanin-based plumages, we constructed the model using locality-specific UV-B values as fixed factor, *B2* for each plumage patch as response variable and sampling site as the random effect. Analyses were carried out in Rstudio ver. 1.2.1335 (R Studio Team 2016).

#### Redundancy analysis and variance partitioning

To explain the proportion of the phenotypic variation (morphology and plumage coloration from the avian perspective) explained by environment, geography and a joint effect, we performed redundancy analyses (RDA), a constrained technique that is a multivariate analog of a simple linear regression [87]. Studies have demonstrated that RDA has greater power than the Mantel test in terms of detecting relationships in autocorrelated data [88,89]. We first calculated variance inflation factors (VIFs) for our explanatory variables in order to avoid collinearity, using the function *vif*.*cca* [90]. After excluding variables with a VIF greater than 10, we performed a stepwise model selection using the function *ordistep* in *vegan* [91] to identify the variables that best explained phenotypic variation, followed by a significance test *anova*.*cca* with 10,000 permutations in both directions. The final environmental uncorrelated variables (tree cover, NDVI_r, NDVI_d, elevation, mean diurnal range (Bio2), mean temperature of driest quarter (Bio9), precipitation of wettest month (Bio13), precipitation of driest month (Bio14), precipitation seasonality (Bio15), precipitation of warmest quarter (Bio18), precipitation of coldest quarter (Bio19), ET_d, and ET_r were used as explanatory variables in a series of redundancy (RDA) and partial redundancy (pRDAs) analyses. With RDA, it is possible to compute the variance between two sets of variables (e.g. morphology and environment) while controlling for the effects of a third set of covariables (e.g. geography). With pRDA, the independent and joint effects of multiple sets of predictor variables can be separated (e.g. [8,9]) and the proportion of phenotypic variance explained by the pure effects of environment, geography, and the interaction between these, can be quantified [92]. To accomplish this, three separated RDA were performed: i) a full RDA model with environmental and geographic variables (geographic coordinates) as explanatory variables; ii) a partial RDA (pRDA1) controlling for the effect of geography (i.e. a purely environmental model); and iii) a partial RDA (pRDA2) controlling for the effect of the environment (i.e. a purely geographic model). The significance of each RDA was estimated with 1000 permutations. The adjusted coefficient of multiple determination (*R*^2^_*adj*_) was calculated for all models [93]. Environmental and phenotypic data were log transformed and then centered and standardized prior to RDA analysis. Explanatory variables also included spatial variables defined using the principal coordinates of neighborhood matrices (PCNM), which were calculated from the geographic coordinates in decimal degrees [94]. The bases of phenotypic data (morphology and color from the avian perspective) were tested independently. These analyses were carried out in R using the *vegan* package [91].

#### Niche conservatism tests

We used occurrence records for all of the distribution of *T*. *sinaloa* from the Global Biodiversity Information Facility (GBIF 2020), accessed from R via *rgbif* (https://github.com/ropensci/rgbif; taxon key: 7341103; https://doi.org/10.15468/dl.z5r355). Data cleaning was performed in RStudio and included filters of: records without georeference information (*dplyr*: [95]; *CoordinateCleaner*: [96]), duplicates in a radius of 0.01666 equivalent to 2 km^2^ (*nichetoolbox*: [97]), records that fell outside of an altitudinal range from 0–2000 m asl (*raster*: [76]), records that fell outside the Neotropical regions where the species occurs (provinces: Sierra Madre Occidental, Trans-Mexican Volcanic Belt, Sierra Madre del Sur, Pacific Lowlands, Balsas Basin; [98,99]), since these could be presumptive errors. Following data cleaning we obtained a total of 909 unique occurrence records.

We estimated four indices, using current bioclimatic variables from the WorldClim database ([75]), following Cortés-Ramírez et al. [100]. The indices were obtained by PCA, retaining the first principal component for each datum from the occurrence records (S1 Fig). Indices were calculated from the following variables: **1) Temperature variation** (BIO1: annual mean temperature, BIO5: max temperature of warmest month, BIO6: min temperature of coldest month, BIO8: mean temperature of wettest quarter, BIO9: mean temperature of driest quarter, BIO10: mean temperature of warmest quarter, BIO11: mean temperature of coldest quarter), **2) Temperature range/seasonality** (BIO2: mean diurnal range, BIO3: isothermality, BIO4: temperature seasonality, BIO7: temperature annual range), **3) Variation of precipitation in the rainy season** (BIO12: annual precipitation, BIO13: precipitation of wettest month, BIO16: precipitation of wettest quarter, BIO18: precipitation of warmest quarter), and **4) Variation of precipitation in dry season** (BIO14: precipitation of driest month, BIO15: precipitation seasonality, BIO17: precipitation of driest quarter, BIO19: precipitation of coldest quarter).

To understand the role of ecological conditions in niche conservatism, we compared niche overlap between subspecies (*cinereus*, *sinaloa*, *russeus*) using an ordination approach, applying the Schoener’s D index [101], as well as equivalency and similarity tests [102]. As described in Broennimann [103], this approach was performed in three steps: 1) calculating the density of occurrences records and environmental factors (first PC of four previous indices) along the environmental axes of a multivariate analysis, 2) measuring niche overlap along the gradient of this multivariate analysis, 3) performing statistical tests of equivalency and similarity to compare the empirical observed distributions of Schoener’s D to 100 simulated values [102,103]. These analyses were implemented using the R package *ecospat* [104] which allows the performance of niche equivalency and similarity tests assessing the significance of a measured niche difference against null model niches taken randomly within a given background area [104]. We defined our study area (background) based on the known geographic distribution of *T*. *sinaloa* (accessible area for the species = M). The accessible area calibration was performed using the *ellipsenm* R package [105], defining a concave polygon delimiting occurrence points with a buffer distance of 75 km.

Niche overlap metrics range from 0 (no overlap) to 1 (complete overlap). Following Röder and Engler [106], the Schoener’s D results suggest: 0–0.2 scores (no or limited overlap), 0.2–0.4 (low overlap), 0.4–0.6 (moderate overlap), and 0.6–0.8 (high overlap). Niche equivalency test determine whether the niches occupied by a pair of subspecies are equivalent. If the observed value of D falls within the density of 95% of simulated values, the null hypothesis of niche equivalency cannot be rejected. The similarity test addresses whether the environmental niche occupied by one subspecies is more similar to that of the other subspecies than would be expected by chance. If the observed overlap value is greater than 90% of the simulated values, the niches occupied by both subspecies are more similar than would be expected by chance. Hypotheses of niche conservatism based on niche similarity tests are accepted when the observed D values for niche overlap differ significantly (*P* < 0.05) from simulated overlap values [103]. All analyses were performed in Rstudio ver. 1.2.1335 (R Studio Team 2016).

## Results

### Morphology

**Fig 2:**
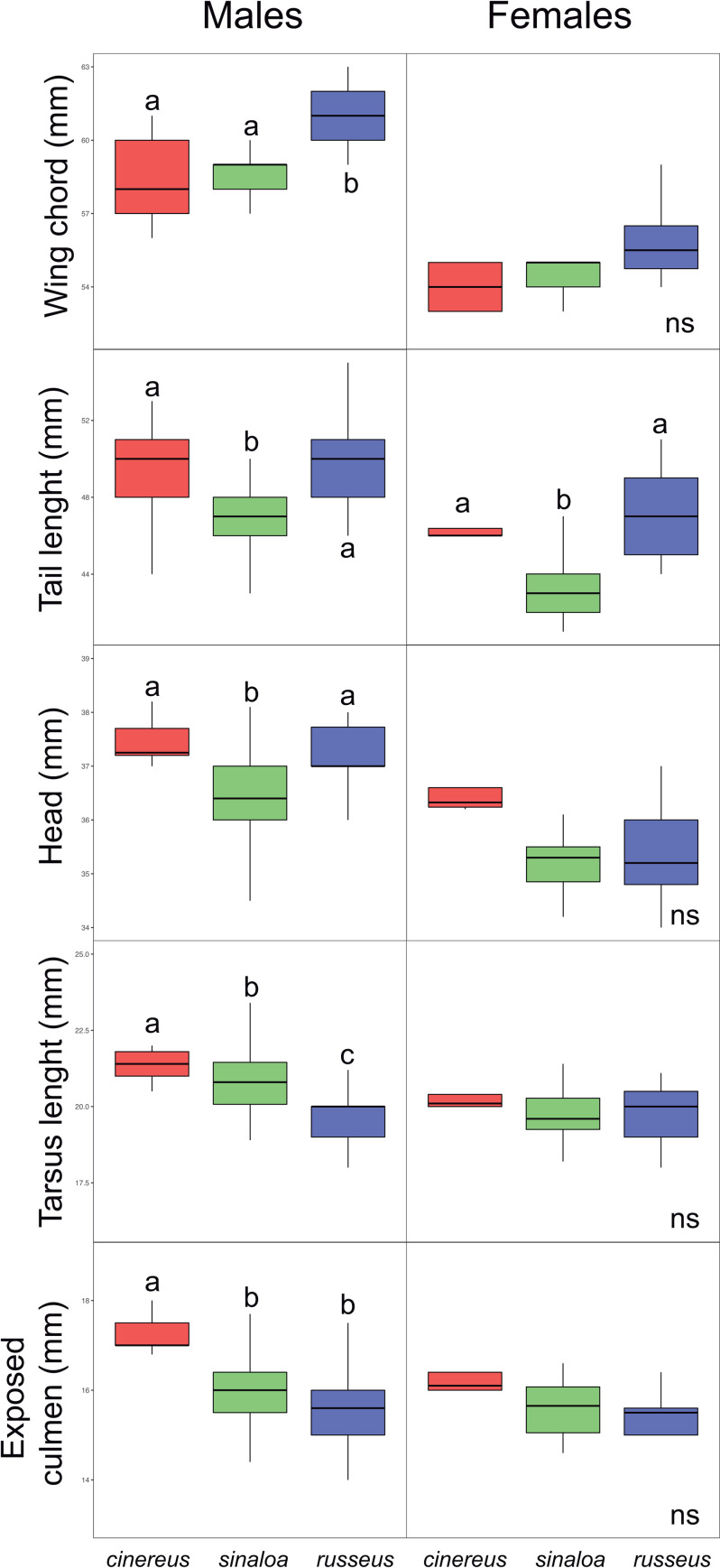
Boxplots from morphological traits within sexes among subspecies of *Thryophilus sinaloa* from field measurements. Boxplots show the percentiles of 25%, 50% (median), and 75%, upper and lower whisker show quartiles of 25%. Significant differences among subspecies are shown with different letters.

All samples collected in the field were successfully and correctly sexed by molecular techniques. The band pattern we obtained was consistent with that reported in Griffiths et al. [68]; i.e., double bands for females and a single band for males. The correct sex assignment was consistent with the gonad descriptions of tissues samples from museum specimens. In general, the three subspecies showed males to be larger than females in all of the measured traits **(**S2 Table). Considering both the field and museum measurements, all between-sex comparisons were statistically significant except for tail length, head and exposed culmen in *cinereus* subspecies, and tarsus as well as exposed culmen in *russeus*, after Bonferroni corrections (S2 Table). Considering the field measurements only, the exposed culmen was also not statistically different in the *sinaloa* subspecies (S2 Table). Kruskal-Wallis comparisons between males among subspecies showed significant differences in all studied traits after Bonferroni corrections, considering both museum and field measurements as well as field measurements only (Table 1 and Fig 2). Conover-Iman post hoc tests showed *russeus* as having the longest wings and longer tails than *sinaloa*, *cinereus* having larger heads than *sinaloa*, as well as a longer tarsus and culmen. In females, significant differences among subspecies were only detected for tail length and head (Table 1), considering both museum and field measurements after Bonferroni corrections. Conover-Iman post-hoc tests showed *cinereus* as having larger heads than *sinaloa* and *russeus*, and *sinaloa* having shorter tails than *cinereus* and *russeus*. Considering field measurements only, tail length was the only trait that maintained significant differences (Table 1 and Fig 2).

**Table 1:** Intrasexual comparison of morphological traits among subspecies (*cinereus*, *sinaloa and russeus*).

	Museum and Field measurements			Field measurements		
	n = 155			n = 120		
***Males***	**X** ^**2**^	***df***	***P*-value**	**X** ^**2**^	***df***	***P*-value**
Wing chord	38.10	2	**<0.001**	36.44	2	**<0.001**
Tail length	36.15	2	**<0.001**	38.07	2	**<0.001**
Head	23.80	2	**<0.001**	24.45	2	**<0.001**
Tarsus length	24.58	2	**<0.001**	33.14	2	**<0.001**
Exposed culmen	22.46	2	**<0.001**	20.11	2	**<0.001**
***Females***		n = 47			n = 27	
Wing chord	6.80	2	ns	2.23	2	ns
Tail length	24.27	2	**<0.001**	15.2	2	**<0.01**
Head	12.78	2	**<0.01**	8.76	2	ns
Tarsus length	2.20	2	ns	2.10	2	ns
Exposed culmen	7.14	2	ns	4.65	2	ns

Comparisons were performed considering museum and field measurements together and only field measurements. Significant comparisons between subspecies are highlighted in bold after Bonferroni corrections. ns = non-significant comparisons.

### Plumage reflectance

**Fig 3:**
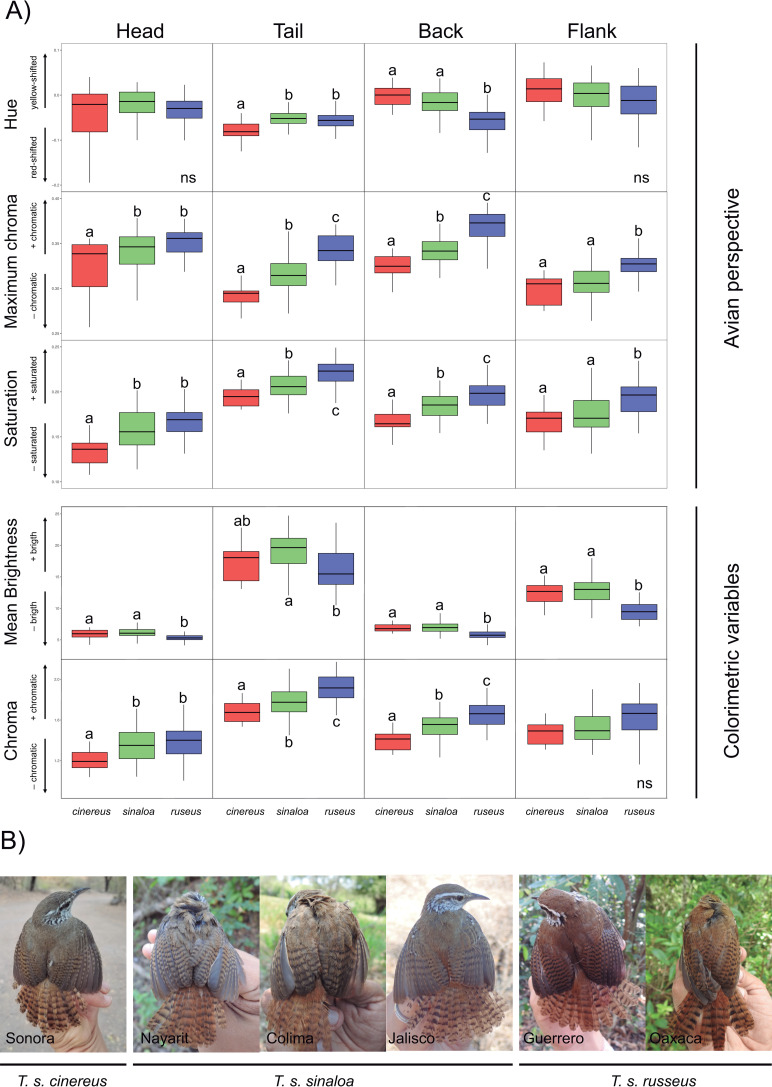
Plumage variation among subspecies. **A.** Boxplots of reflectance variables from the avian perspective (hue, maximum chroma and saturation) and colorimetric variables (mean brightness and chroma) among *Thryophilus sinaloa* subspecies. Boxplots show the percentiles of 25%, 50% (median), and 75%; upper and lower whisker show quartiles of 25%. Significant differences among subspecies are shown with different letters. **B.** Photographs showing dorsal plumage variation among subspecies of the Sinaloa Wren.

No significant differences were detected using the Wilcoxon-Mann-Whitney tests to compare intersexual variation in the variables collected with reflectance spectrometry (hue, maximum chroma, saturation, brightness and chroma; results not shown), therefore the sexes were pooled together in subsequent analyses. From the avian perspective, we found significant differences among subspecies in all color variables for all plumage patches, except for hue on the head and flanks (Table 2). The *cinereus* and *sinaloa* subspecies showed back plumages with higher hue values, or more yellow-shifted color than *russeus* that showed more red-shifted color (Fig 3). However, the opposite pattern was found in the tail (Fig 3). Differences in maximum chroma among subspecies showed the same pattern in all of the studied plumage patches (Table 2 and Fig 3). The *cinereus* subspecies showed a dull plumage, while that of *russeus* was more vivid or intense (i.e. more chromatic) and *sinaloa* presented intermediated values (Fig 3). Finally, *russeus* showed more saturated plumage (higher values of *r vec*) while that of *cinereus* was less saturated (Fig 3). From the colorimetric variables, we found significant differences among subspecies when comparing mean brightness (*B2*) and chroma (*S8*) in all studied patches, except for chroma in flanks (Figs 3 and S2). The *cinereus* and *sinaloa* subspecies showed brighter plumage compared to *russeus*, while *sinaloa* and *russeus* presented more chromatic plumages (i.e. more intense or vivid).

**Table 2:** Comparison of plumage reflectance data between subspecies of the Sinaloa Wren from the avian perspective and from colorimetric variables.

	X^2^	*df*	*P*-value
***Avian perspective***			
**Hue (*hue theta*)**			
Head	5.64	2	ns
Tail	15.76	2	**<0.001**
Back	41.48	2	**<0.001**
Flank	2.74	2	ns
**Maximum chroma (*r max*)**			
Head	14.35	2	**<0.001**
Tail	58.59	2	**<0.001**
Back	44.47	2	**<0.001**
Flank	28.74	2	**<0.001**
**Saturation (*r vec*)**			
Head	19.12	2	**<0.001**
Tail	36.97	2	**<0.001**
Back	35.20	2	**<0.001**
Flank	17.53	2	**<0.001**
***Colorimetric variables***			
**Mean Brightness (*B2*)**			
Head	24.97	2	**<0.001**
Tail	21.66	2	**<0.001**
Back	33.27	2	**<0.001**
Flank	5.35	2	**<0.001**
**Chroma (*S8*)**			
Head	14.51	2	**<0.001**
Tail	35.51	2	**<0.001**
Back	36.24	2	**<0.001**
Flank	13.63	2	ns

Significant comparisons are highlighted in bold after Bonferroni corrections.

Discriminant comparisons in plumage color among pairs of subspecies showed a general pattern of color discrimination in all of the studied patches (i.e. ΔS < 1 JND, S3 Fig), except for flanks between *cinereus* and *sinaloa*, and for head between *russeus* and *sinaloa* subspecies (S3 Fig). Greater values of color discrimination were recorded between subspecies at the extremes of the distributional range (*cinereus* and *russeus*), with tail color patches being the most discernible among these subspecies (i.e. ΔS = 3.7 JND). Similar JND mean values were recorded for *cinereus* vs. *sinaloa* and for *russeus* vs. *sinaloa* comparisons. For the *sinaloa* and *russeus* subspecies, all plumage patches were discernible between subspecies, apart from the head plumages (S3 Fig).

### Environment and geography explaining phenotypic variation

The GLMM simplified models showed a significant and positive relationship of elevation, annual mean temperature and annual precipitation with wing chord and tail length (i.e. longer wings and tails at higher elevations, and in warmer and wetter areas, Table 3), as well as between latitude and tail length. A positive relationship of tree cover and latitude with culmen length was also found (i.e. longer culmen in more dense habitats and at higher latitudes), while for tarsus length, a positive and significant association was found with latitude (i.e. longer tarsus at higher latitudes, Table 3). The effect of sex was significant in all previous models. For plumage, we found a positive relationship between annual mean temperature and plumage brightness on the back, flanks and tails (i.e. brighter plumages in warmer habitats; Table 3). Back and flank plumage brightness was negatively associated with annual precipitation and back and tail plumage was positively associated with evapotranspiration during dry the season (i.e. darker plumages in more humid conditions). Plumage brightness on the back and tails was positively associated with NDVI during the dry season (i.e. darker plumages in greener areas). Head plumage brightness was not shown in the simplified model, since none of the predictor variables were found to be significant. UV-B radiation was significantly and negatively associated with plumage brightness in heads (*t* value = -2.23, *P* < 0.05) and backs (*t* value = -2.5, *P* < 0.05) (i.e. darker head and back plumages at more UV-B radiation, S3 Table). Not significant association was found in flank and tail plumages brightness and UV-B (S3 Table). Full GLMMs are shown in S3 Table.

**Table 3:** Mixed effects models over phenotypic traits of *Thryophilus sinaloa*.

	Estimate	Std. Error	t value
***Morphology***			
**Wing chord**			
(Intercept)	4.13e+01	5.60e+00	7.36
Elevation	2.65e-03	7.66e-04	3.46***
Annual mean temperature	4.35e-01	2.18e-01	1.99*
Annual precipitation	1.36e-03	6.20e-04	2.19*
Sex	4.43e+00	3.10e-01	14.27***
**Tail lenght**			
(Intercept)	3.46	12.1919	0.284
Elevation	0.005	0.0013	4.149***
Annual mean temperature	1.05	0.3826	2.754**
Annual precipitation	0.003	0.0011	2.770**
Latitude	0.38	0.1288	2.961**
Sex	3.96	0.4100	9.662***
**Exposed culmen**			
(Intercept)	12.30	1.0718	11.484
Tree cover	0.01	0.0059	1.692*
Latitude	0.10	0.0355	2.816**
Sex	0.67	0.1350	4.993***
**Tarsus length**			
(Intercept)	18.14	0.9016	20.128
Latitude	0.09	0.0430	2.240*
Sex	0.80	0.1651	4.855***
***Plumage brightness***			
**Back**			
(Intercept)	3.77	2.1984	1.715
Annual mean temperature	0.23	0.0934	2.481*
Annual precipitation	-0.001	0.0005	-2.221*
Evapotranspiration dry	0.05	0.0164	3.337***
NDVI dry season	-4.07	1.4181	-2.871**
**Flank**			
(Intercept)	3.35	5.9769	0.561
Annual mean temperature	0.47	0.2343	2.021*
Annual precipitation	-0.003	0.0014	-2.420*
Evapotranspiration dry	0.05	0.0385	1.475
**Tail**			
(Intercept)	-2.35	10.1941	-0.231
Annual mean temperature	1.21	0.4378	2.776**
Evapotranspiration dry	0.18	0.0807	2.277*
NDVI dry season	-19.90	6.7216	-2.961**

Variables kept in the final model after backward elimination are shown. *** Significant values < 0.001, ** <0.01, * <0.05 are highlighted in bold.

The full RDA model for the combined effect of geographic and environmental explanatory variables on phenotypic variation was significant for both morphology and color from the avian perspective (Table 4). The partial RDA (pRDA1) for the association between environmental variables (purely environment) and morphology controlling for the effect of geographic distance was statistically significant. The proportion of morphological variation explained by the environment (13%) was higher than that explained by geography (8%) (Table 4). The pure effect of environment and geography explaining plumage variation from the avian perspective was not significant. Most of the variation in both phenotypic groups of variables was explained by the joint effect of environment and geography (morphology: 79% and color from the avian perspective: 95%, Table 4). The most important environmental variables that best explained phenotypic variation included different precipitation related variables, temperature, evapotranspiration, elevation and NDVI (S4 and S5 Tables and S4 Fig).

**Table 4:** Results of the redundancy analysis (RDA) for the association between phenotypic variation (morphology and color from the avian perspective), geographic and environmental variables for *Thryophilus sinaloa* populations.

	Inertia	Proportion	*R*^2^_adj_	*P-value*
***Morphology***				
Full model: geography and environment	5.00	1.00	0.20	**0.001**
Pure environment (pRDA1)	0.65	0.13	0.10	**0.001**
Pure geography (pRDA2)	0.40	0.08	0.05	**0.001**
Joint environment/geography	3.94	0.79	0.04	NA
***Color from avian perspective***				
Full model: geography and environment	11.41	1.00	0.21	**0.001**
Pure environment (pRDA1)	0.31	0.03	0.00	0.241
Pure geography (pRDA2)	0.26	0.02	1.00−^05^	0.462
Joint environment/geography	10.84	0.95	0.21	NA

Full model and two partial analyses are shown: pRDA1 (effect of environmental variables while controlling for geographic distance) and pRDA2 (effect of geographic distance while controlling for environmental variation). Variance (inertia) and the proportion of total variance explained by each model (proportion) are indicated in each case. The proportion of variance explained by both geographic and environmental effects is also indicated (joint environment/geography).

### Niche comparison tests

**Fig 4:**
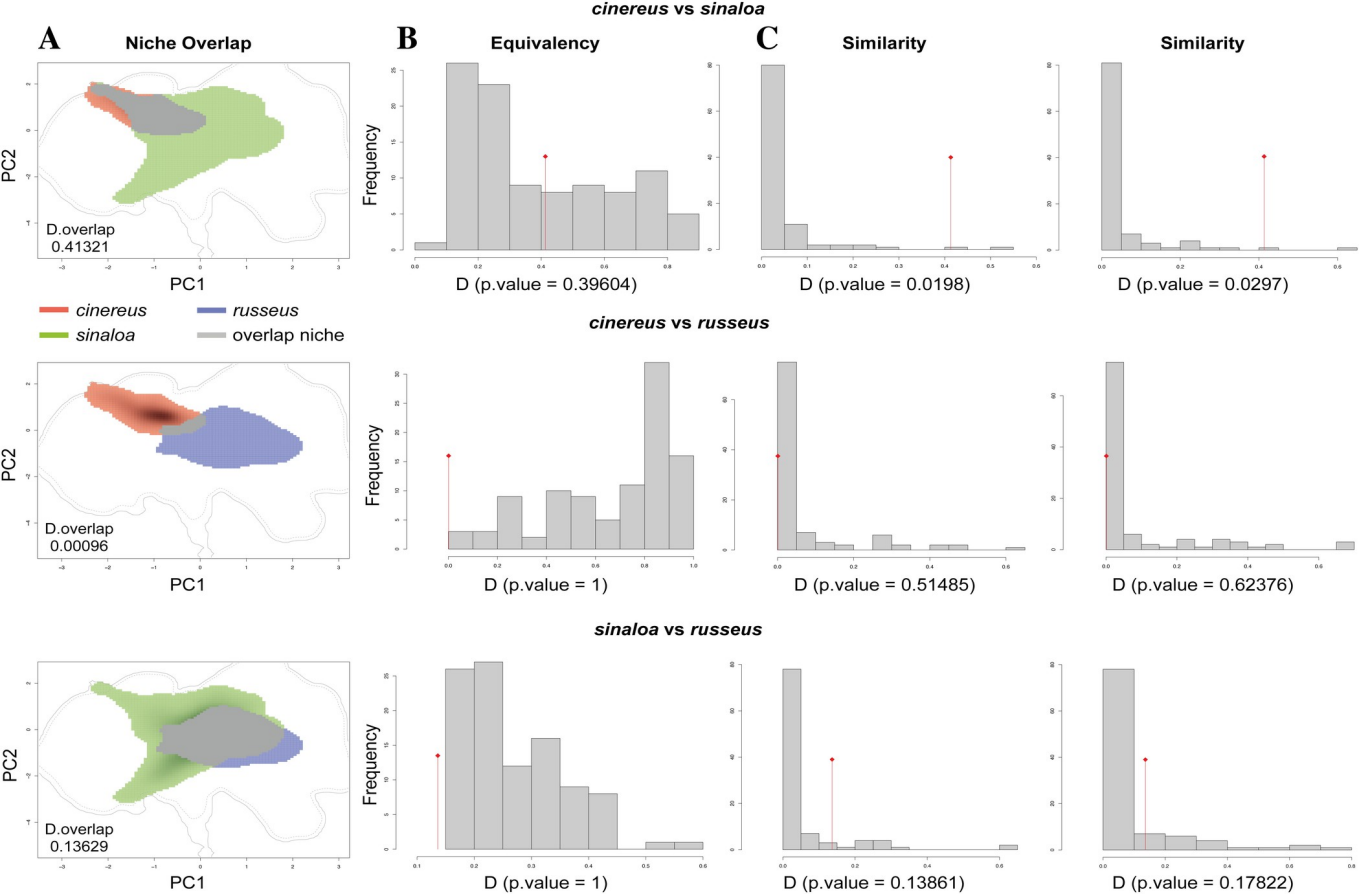
Graphical results of niche overlap, equivalence and similarity tests in environmental space for Sinaloa Wren. **A.** Graphical results of environmental niche overlap between pair of subspecies (gray areas) obtained from an ordination approach; **B.** Niche equivalence test between pair of subspecies, if the observed D values falls within the 95% density of simulated values, the null hypothesis of niche equivalency cannot be rejected; **C.** Similarity test between pair of subspecies in both directions, niche conservatism is accepted when the observed D values for niche overlap are significantly different (*P* < 0.05) from simulated values of overlap.

Factor loading and proportions of variance for the first component of the climatic indices are summarized (S6 Table). According to our PCA analysis, based on density records and the environmental factors (first PC of four previous indices), the first two components explained 46.32% and 33.39% of variation, respectively, and were used to represent the environmental niche for each subspecies in a two-dimensional gradient. According to the niche overlap D values between the subspecies pairs comparisons, moderate niche overlap is suggested for *cinereus* vs. *sinaloa* (D = 0.41321), and no or limited overlap for *cinereus* vs. *russeus* (D = 0.00096), and for *sinaloa* vs. *russeus* (D = 0.13629; Fig 4A). The equivalency test based on comparison between observed D values and simulated values showed that the niches occupied by the *cinereus* and *sinaloa* subspecies are more equivalent than would be expected by chance (Fig 4B). The opposite pattern was observed in the equivalency test for the *cinereus* vs. *russeus* and *sinaloa* vs. *russeus* subspecies comparisons (Fig 4B). Niche conservatism, based on similarity tests, was accepted for the *cinereus* and *sinaloa* niche comparison in both directions (Fig 4C). No niche conservatism was detected in the *cinereus* vs. *russeus* and *sinaloa* vs. *russeus* comparisons in either directions (Fig 4C), suggesting niche divergence.

## Discussion

Using an integrative approach, we studied phenotypic variation in the Sinaloa Wren along its distribution in the TDF of the Mexican Pacific slope, in the absence of evident geographic barriers. According to the three objectives of our study, our results showed: 1) that the subspecies of the Sinaloa Wren (*cinereus*, *sinaloa* and *russeus*) exhibited significant phenotypic differences in morphology and plumage coloration, 2) that ecogeographic rules partially support the relationship between phenotypic and environmental variation, that the combined effect of geography and environment explained the major proportion of all of the studied phenotypic variation and that environmental variables explained a slightly greater proportion of the morphological trait variation than geography alone, and 3) there was moderate niche overlap between subspecies of the north and center of the distribution (*cinereus* and *sinaloa*), suggesting niche conservatism, and no or limited overlap between *cinereus and sinaloa* (north and center of the distribution) against *russeu*s (south of the distribution) subspecies, suggesting niche divergence. This evidence indicates that, even when the TDF is a relatively continuously distributed habitat, variation in environmental conditions is important for promoting and maintaining morphological differentiation between populations. For plumage coloration, the combined effect of environment and geographic isolation could be the primary factor shaping color variation in the Sinaloa Wren.

### Phenotypic variation

As previously recorded in Guallar et al. [107], our data support significant sexual dimorphism in the morphology of this species, with males generally presenting larger size than females in all of the traits studied within the subspecies. This sexual dimorphism was not maintained in the plumage coloration of the studied feather patches, since both sexes presented similar coloration from the avian perspective and according to the colorimetric variables. Despite finding significant differences in the morphological traits measured between subspecies, there was no clear direction in size differentiation. However, the differences among subspecies in specific morphological traits highlighted in our results suggest adaptation to local conditions (see below).

A general pattern was observed in plumage coloration among the subspecies that agreed with the subspecies descriptions. At the northern limit of distribution, the *cinereus* subspecies has yellowish, duller, brighter and less saturated plumages. In contrast, *russeus* at the southern limit of distribution has reddish, darker and more vivid plumages (more saturated). Moreover, the chromatic differences based on avian discrimination modelling among the subspecies were greater than the theoretical discrimination in almost all of the studied patches, with the highest values found between subspecies at the extremes of the distribution (*cinereus* and *russeus*). This suggests that individuals from the three subspecies could discriminate members of the other subspecies through plumage coloration. Song differentiation was also detected among subspecies during fieldwork (A. M. and C. G. personal observations); however, specific studies of vocal variation as well as song recognition between subspecies are necessary in order to assess whether song plays a role as a premating mechanism of reproductive isolation (e.g. [108]).

### Phenotypic variation patterns explained by the environment

Our analyses to examine the relative importance of environmental factors explaining morphology and plumage color differentiation suggested that the combined effect of geography and environment explained most of the phenotypic variance observed in the Sinaloa Wren. The pure effect of environment explained a slightly higher proportion of the phenotypic variance in morphology than geography alone. In contrast, only the combined effect of environment and geography were significant in explaining variation in plumage color; e.g., at the extremes of the latitudinal gradient, where the species is distributed along the TDF, contrasting environmental conditions are presented. According to our RDAs, variation in precipitation across the TDF rather than temperature is one of the most important factors explaining phenotypic variation. This is in accordance with the variation of annual mean precipitation, which ranges from 690 mm/yr in Sonora to 1460 mm/yr in Oaxaca, representing the two most extreme localities within the distribution of the Sinaloa Wren.

We found that longer wings were related to warmer areas, which contradicts Bergmann’s rule, using wing chord as a proxy of body size. This rule explains that larger bodies are an advantage in more severe climates, since they irradiate less body heat per unit mass and therefore keep the individual warmer in colder climates [15]. Zink & Remsen [109] reviewed geographic body size variation in 92 North American bird species, indexed by wing length. From these, 42% of the studies agreed with Bergmann’s rule, while 29% contradicted the rule’s expectations, suggesting that the empirical basis for the existence of Bergmann’s rule is weak, at least for North American birds.

According to Allen’s rule, shorter appendages are expected to be related to colder climate conditions in order to reduce thermoregulatory costs. However, our data did not support this trend since no significant relationship was found between temperature and appendage length (tarsus or culmen). This ecogeographic rule has been largely ignored in the literature, and the results of the few studies that exist report patterns that only partially support the rule (e.g. [110] for culmen), and others that do not support it at all (e.g. [111]). Along the distribution of the Sinaloa Wren, differences in temperature are small and are probably insufficient to reflect these ecogeographic rules, since the organisms at this scale do not really experience harsh climates in terms of temperature.

Although our results did not support these rules, other morphological patterns could suggest ecological adaptations to specific habitat conditions. We found a significant positive relationship between wing and tail length and the parameters of elevation, annual mean temperature and annual precipitation. Longer wings and tails at higher altitudes have been suggested as an adaptation to increase flight efficiency and lift balance under more challenging air conditions, but proper studies are required in order to test this specific relationship. Individuals from the subspecies *russeus*, which had the longest wings and longer tails than *sinaloa*, were generally captured at higher altitudes than the other subspecies. Differences in altitude among sampling sites where the subspecies were collected varied from 366 to 482 m asl in *cinereus*, from 22 to 1404 m asl in *sinaloa* and from 515 to 1598 m asl in *russeus*. Moreover, in the habitats where *russeus* was collected, the vegetation was greener (due to higher precipitation), and the understory of the region is not as dense or intricate as it is in the rest of the distribution of the species. This probably creates a more open habitat that influences longer wings in order to increase efficiency in direct flight [112]. On the other hand, the longer culmens found at the higher latitudes inhabited by *cinereus* could be associated with larger sized insect prey, but shorter wings could be associated with improved maneuverability in flight performance in a more intricate understory, a typical characteristic of the TDF in the driest areas, and a pattern expected in denser environments [112]. In addition, the longer tarsus found in *cinereus* in Sonora could be associated with the more intricate pattern of the understory that offers more perching options. Grant [113] has suggested that longer tarsi found in some island species may also be linked to greater ecological versatility.

Plumage brightness in most of the studied plumage patches was associated negatively with annual precipitation and NDVI and positively with evapotranspiration, which supports Gloger’s rule. Evapotranspiration measures the loss of humidity from the land surface along with vegetation transpiration. Therefore, higher levels of evapotranspiration results in dryer habitats. Darker plumages in *russeus* were associated with more humid habitats at the southern limit of the distribution (annual mean precipitation = 1500 mm/yr on average), in contrast to the brighter plumages in *cinereus* at the northern limit of the distribution where the annual mean precipitation is less than half the values of the southern distribution (annual mean precipitation = 690 mm/yr on average; Fig 1). This part of the prediction of the Gloger’s rule (darker plumage in rainy climates) has been widely supported, at both intraspecific [114,115] and interspecific [34,35,115,116] level. Furthermore, at continental scale (e.g. [31]), the relationship between humidity and darker coloration has been strongly supported in North American birds: from 52 studies, 96% display the trend predicted by Gloger’s rule while only 4% do not [109]. However, plumage brightness was also positively associated with annual mean temperature, which contradicts Gloger’s rule that predicts darker colorations in warmer climates. The prediction that animals should be darker in warmer areas has received little support [31]; indeed, the opposite pattern has generally been detected (e.g. [31,114,117]), supporting Bogert’s rule that suggests the thermoregulatory advantages of this pattern. In the Sinaloa Wren, higher temperatures were found within the distribution of the *sinaloa* subspecies with brighter plumages, along with *cinereus*. These results agree with recent studies that found support for Gloger’s rule exclusively related to precipitation but not to temperature, suggesting that this rule could be re-defined in terms of precipitation effects alone [31,117].

Several explanations have been proposed for Gloger’s rule. It has been suggested that darker plumages increase resistance against feather degrading bacteria [118], since the increased melanization acts to make feathers more resistant to these bacteria, or confers a thermoregulatory advantage depending on the degree of pigmentation [119]. However, the association of darker plumages with humidity has also been suggested to be a manifestation of background matching to reduce detectability, while humidity per se has little direct influence [109,120,121]. Endler [121] states that animals inhabiting dark and closed environments should be darker compared to those inhabiting open areas with higher ambient light, a supported explanation to enhance crypsis in birds. This pattern is consistent with the so-called complex version of Gloger’s rule which predicts that in warm/humid habitats animals tend to be gray or black since eumelanins prevail, while in warm/dry habitats animals tend to be brown or rufous since pheomelanins prevail (e.g. [116,117]). In the Sinaloa wren, the habitat of the *russeus* subspecies is found at higher altitudes in a transition zone between the TDF and pine-oak forest, in which the trees do not lose all of their foliage, and therefore the evergreen vegetation remains green during the dry season (NDVI dry = 0.67), which would be more similar to a shaded forest condition, compared to the rest of the distribution of the species (NDVI dry for *cinereus* = 0.36 and for *sinaloa* = 0.58). This is consistent with the notion that plumage coloration evolves to maximize crypsis relative to the environment [120–122], in which brighter, less saturated plumages (*cinereus*) match more open drier/grayer habitats at the northern limit of the distribution, where the vegetation completely loses its foliage during the dry season, while darker more saturated plumages (*russeus*) match the more closed humid/green habitats where the vegetation preserves much of its foliage even during the dry season.

In addition to all these explanations to understand the relationship between the variation in plumage coloration and the environment, recent studies have demonstrated the possible influence of other factors in explaining melanin-based pigmentation. These include levels of UV radiation, natural ground radioactivity, as well as habitat and dietary factors, that may represent a source of oxidative stress or influence the synthesis of melanin, which would in turn affect body condition and ultimately melanin production and be manifested in different plumage colorations [29,35,123,124]. In this study, we only included UV-B radiation, which was negatively associated with plumage brightness in two of the studied patches (head and back). That is, darker plumage colorations were associated with higher levels of UV-B radiation. This pattern has been proposed as a mechanism of protection against the negative effects of solar UV radiation [35] such as oxidative stress as explained above. It is likely that head and back in the Sinaloa Wren could be the body parts mostly exposed and therefore the most relevant regions of the plumage in terms of protection against damage caused by solar UV radiation. Our results agreed with those found in Galván et al. [35] for *Aquila chrysaetos* at a continental scale, where eagles exposed to higher levels of UV-B radiation develop darker plumages phenotypes. Nevertheless, we cannot exclude the effects of natural ground radioactivity as well as diet as important factors influencing the melanin gradient pigmentation in the Sinaloa Wren, however, further studies are needed to explore this.

The niche overlap tests suggested moderate niche overlap and niche conservatism between *cinereus* and *sinaloa* (the northernmost and central subspecies, respectively). This result indicates that these two subspecies occupy similar environmental conditions. However, morphological and plumage color differentiation was supported for this pair of subspecies even when phenotypic differences in feather plumage between this pair of species seems minimal to the naked eye. Overlap and niche divergence were not found in the *cinereus*–*russeus* and *sinaloa*–*russeus* comparisons, suggesting that these pairs of subspecies inhabit different environmental conditions. The *russeus* niche divergence from the other two subspecies is plausible since records from *russeus* subspecies in online databases are rare on the coast (at lower elevations). This was confirmed by fieldwork conducted during this study, when we searched for *russeus* in some parts of the coast in Oaxaca and Guerrero but found no samples. The individuals of this subspecies included in this study were all captured in the ecotone between TDF and pine oak forest at higher elevations. The difference in niche occupancy was also accompanied by the greatest phenotypic differences between *cinereus* and *russeus*, suggesting the importance of environment as a promoter of phenotypic differences.

In contrast with evidence of phenotypic divergence within bird species from montane habitats, such as cloud, pine and pine-oak forests, as a result of drift in isolation by geographic barriers [46,50,125], our findings suggest that ecological barriers are important in terms of promoting phenotypic differentiation among lowland habitats with no apparent geographic barriers to dispersal. However, this is only the first evidence to contribute to our understanding of the mechanisms that promote phenotypic differentiation along the TDF. It is recognized that phenotypic traits variation could also arise via environmental plasticity or through local adaptation as a result of natural selection. The inclusion of genetic data is necessary in order to distinguish between these two mechanisms that could promote phenotypic variation (e.g. [8,21,126,127]). Moreover, the use of neutral and under selection molecular markers (such as single nucleotide polymorphisms: SNPs) could help to separate the effect of drift and local adaptation in phenotypic variation. Other mechanisms such as sexual selection or even competition [128,129] could also explain phenotypic divergence. It is also recognized that the geographic history of a particular region where the species is distributed could deeply influence genetic and phenotypic patterns. The geographic history of the TDFs has been poorly studied and understood (but see [130]); however, past expansions and contractions of the habitat could also explain the phenotypic variation observed at present across the TDF. For example, Castillo-Chora et al. [130] suggested that the biogeographic history of the TDFs of the Pacific Slope has been highly dynamic, resulting in alternating periods of isolation and connectivity that could explain the genetic differentiation patterns found in five co-distributed bird species with a distribution similar to that of *T*. *sinaloa*. We cannot discount the possibility that past geographic climatic changes could also partly explain the phenotypic differences found in the Sinaloa Wren; indeed, niche conservatism in the *cinereus* and *sinaloa* subspecies could be result of a rapid and recent colonization of some part of the distribution. Present and past niche modelling for the distribution of the Sinaloa Wren would be important in order to assess this.

## Conclusions

Our findings suggest that environmental variation (especially that related to precipitation regime and seasonality) across a single ecosystem such as the TDF on the Mexican Pacific slope is important and could be sufficient to promote phenotypic divergence among populations that inhabit this ecosystem. Nevertheless, it is essential to determine whether the patterns of phenotypic variation agree with genetic patterns in the species. This could be important to the differentiation between phenotypic plasticity and adaptation to local environments. Our study provides evidence that contributes to understanding the factors that promote the wide biodiversity and endemism observed in the TDF, a highly threatened ecosystem.

## Supporting information

S1 Fig Sinaloa Wren subspecies distribution. Occurrence records for the distribution of the Sinaloa Wren retrieved from the Global Biodiversity Information Facility (GBIF 2020), used for ecological niche tests. Putative distribution for each subspecies is shown with different colors.
(TIF)

S2 Fig Mean spectra curves representing brightness in feather patches. We show examples of dull (blue), medium (green) and bright (red) feathers for each plumage patches: A) head, B) back, C) flank and D) tail.
(TIF)

S3 Fig Visual representation of color discriminability in avian perceptual space (ΔS) for all plumage patches between subspecies pairs. Threshold for color differentiation is ΔS = 1JND (just noticeable differences). Values below this threshold (green line) are perceived as the same color.
(TIF)

S4 Fig Environment-phenotypic associations (pRDA1) for the Sinaloa Wren. Points represents the projection of each individual on the first two RDA axes. The environmental variables are shown by labeled vectors where arrows indicate the direction of the gradient variation and the length represents the contribution to each axes of the orthogonal projection. A) morphology, B) feather coloration from the avian perspective.
(TIF)

S1 Table Collection localities for the Sinaloa Wren samples examined in this study. Source from which data was obtained is provided, F = field, M = museum.
(DOCX)

S2 Table Measurements of five morphological traits in male and female Sinaloa Wren subspecies (*cinereus*, *sinaloa* and *russeus*). Statistically significant differences between sexes within subspecies are highlighted in bold after Bonferroni corrections *** <0.001, **<0.01. ns = non-significant comparisons.
(DOCX)

S3 Table Mixed effects full models over phenotypical traits of *Thryophilus sinaloa*. *** Significant values < 0.001, ** <0.01, * <0.05 are highlighted in bold, ns = not significant (*P* > 0.05).
(DOCX)

S4 Table Partial redundancy analysis (RDA) model selection showing environmental variables that best explained phenotypical variation (morphology and color from the avian perspective). Akaike Information Criterion (AIC) and *F* statistics were included. *P-values* representing significant differences are shown in bold.
(DOCX)

S5 Table Loadings of environmental variables into the first two axes of pRDA1. Environmental variables explaining phenotypical variation (morphology and color from the avian perspective).
(DOCX)

S6 Table Summary of factor loadings and proportion of variance of the climatic indexes for the first principal component in a PCA. Summary of factor loadings and proportion of variance of the climatic indexes for first principal component in a PCA.
(DOCX)
