# Supplementary figures and images for: Color and morphological differentiation in the Sinaloa Wren (Thryophilus sinaloa) in the tropical dry forests of Mexico: The role of environment and geographic isolation
Source: PLoS One. 2022 Jun 23;17(6):e0269860. doi: 10.1371/journal.pone.0269860 (PMC9223310; doi:10.1371/journal.pone.0269860)

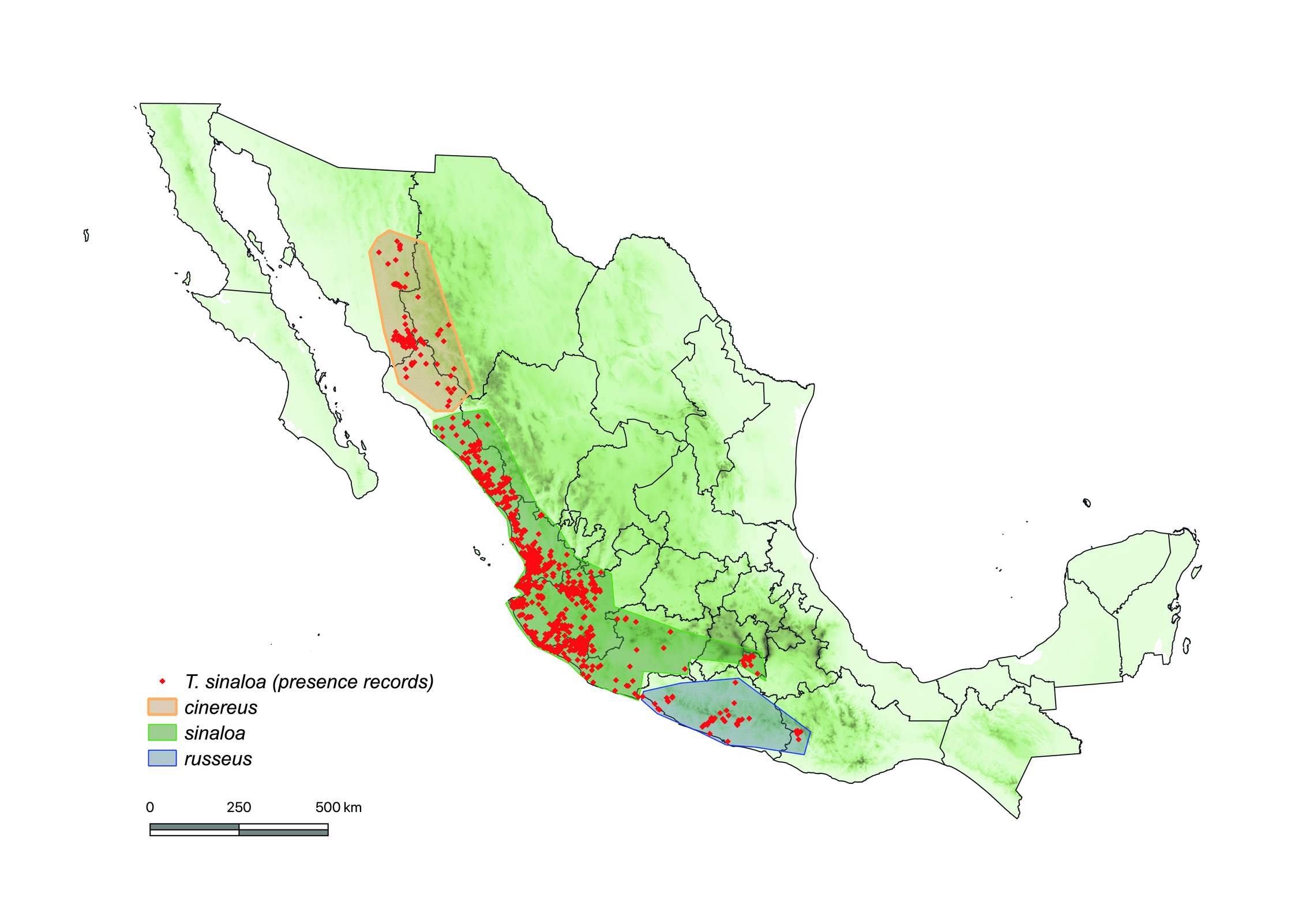

Supplement: S1 Fig — Occurrence records for the distribution of the Sinaloa Wren retrieved from the Global Biodiversity Information Facility (GBIF 2020), used for ecological niche tests. Putative distribution for each subspecies is shown with different colors. (TIF) [file pone.0269860.s001.tif]

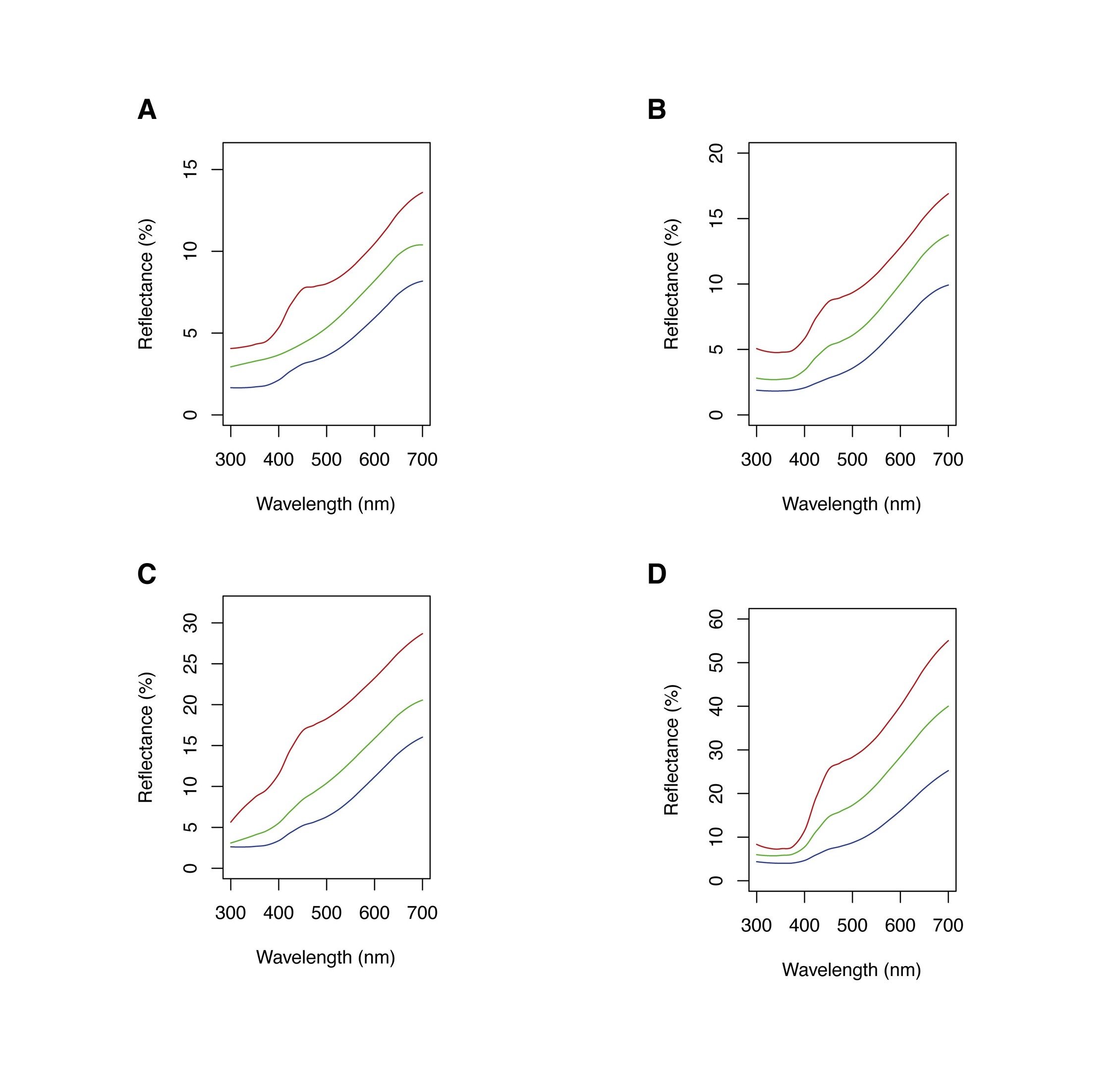

Supplement: S2 Fig — We show examples of dull (blue), medium (green) and bright (red) feathers for each plumage patches: A) head, B) back, C) flank and D) tail. (TIF) [file pone.0269860.s002.tif]

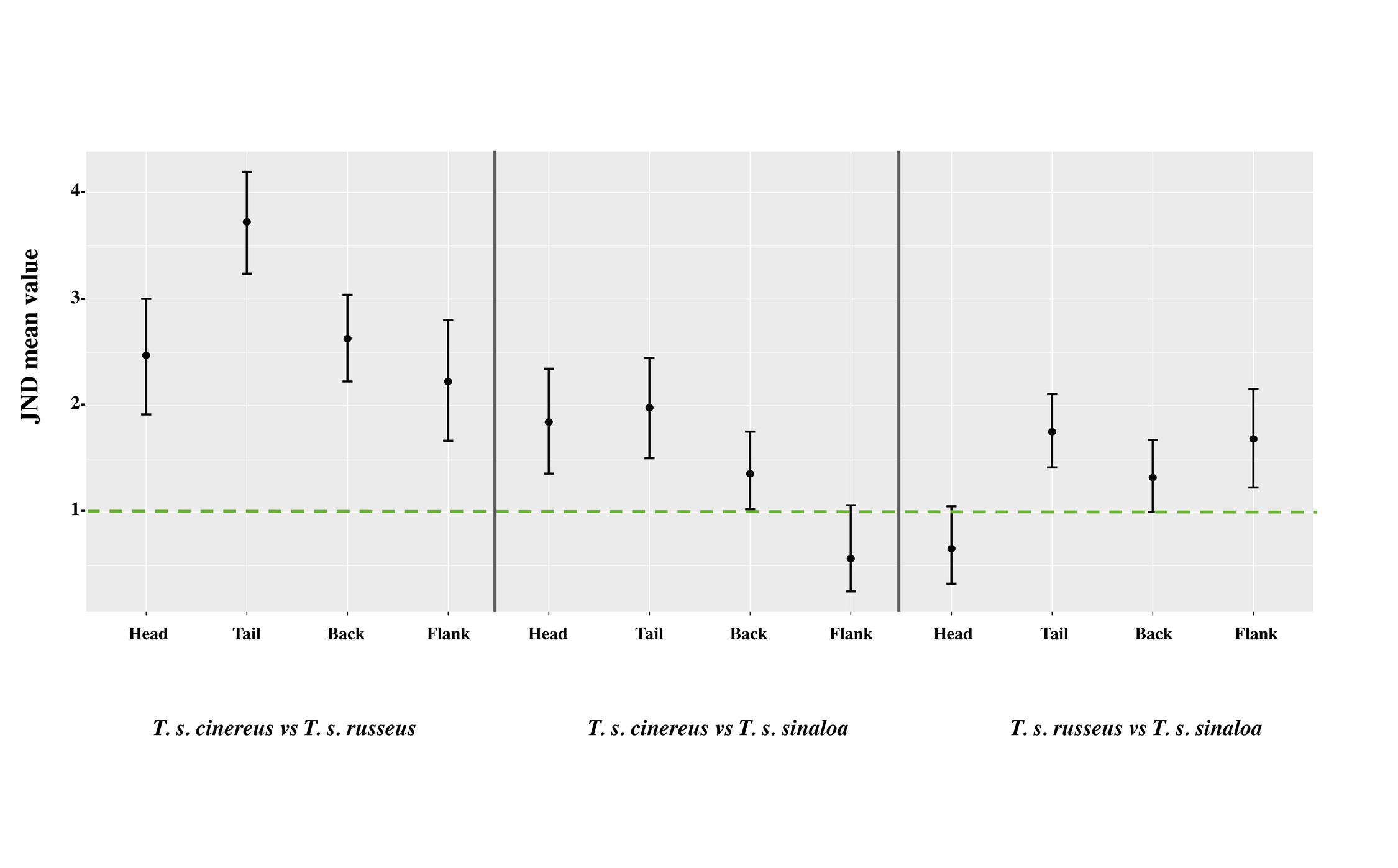

Supplement: S3 Fig — Threshold for color differentiation is ΔS = 1JND (just noticeable differences). Values below this threshold (green line) are perceived as the same color. (TIF) [file pone.0269860.s003.tif]

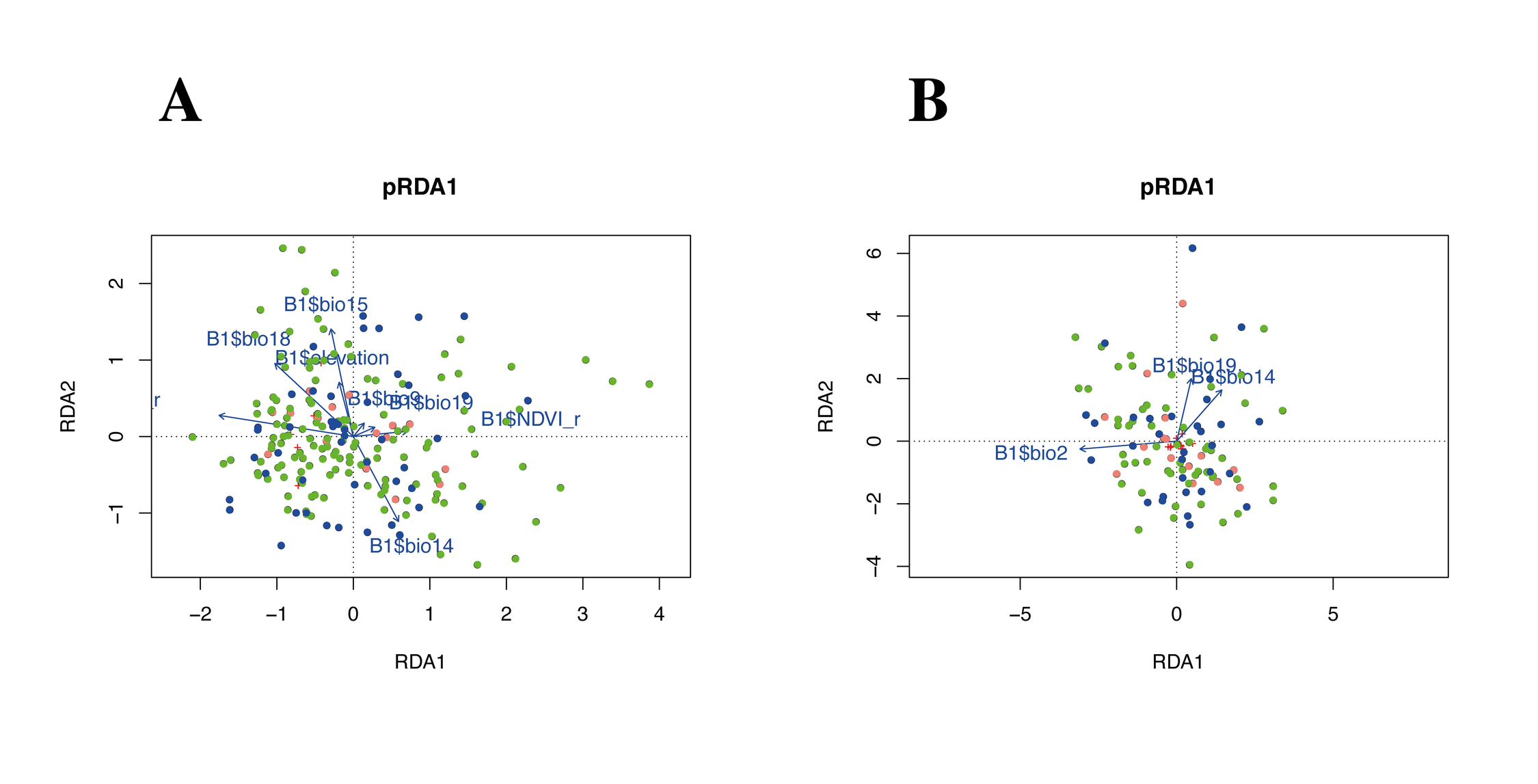

Supplement: S4 Fig — Points represents the projection of each individual on the first two RDA axes. The environmental variables are shown by labeled vectors where arrows indicate the direction of the gradient variation and the length represents the contribution to each axes of the orthogonal projection. A) morphology, B) feather coloration from the avian perspective. (TIF) [file pone.0269860.s004.tif]
