# Supplementary material for: Color and morphological differentiation in the Sinaloa Wren (Thryophilus sinaloa) in the tropical dry forests of Mexico: The role of environment and geographic isolation
Source: PLoS One. 2022 Jun 23;17(6):e0269860. doi: 10.1371/journal.pone.0269860 (PMC9223310; doi:10.1371/journal.pone.0269860)
Supplement: S1 Table — Source from which data was obtained is provided, F = field, M = museum. (DOCX) [file pone.0269860.s005.docx]

**S1 Table. Collection localities for the Sinaloa Wren samples examined in this study.** Source from which data was obtained is provided, F = field, M= museum.

| **Locality** | **State** | **Subspecies** | **Latitude** | **Longitude** | **Elevation (m. asl)** | **Source** |
| --- | --- | --- | --- | --- | --- | --- |
| Arroyo Citorijaqui | Sonora | *cinereus* | 27.0598 | -108.7313 | 460 | F |
| Palo Injerto | Sonora | *cinereus* | 27.0423 | -108.7400 | 425 | F |
| Palmarito | Sonora | *cinereus* | 27.0334 | -108.7851 | 482 | F |
| Arroyo Cuchujaqui | Sonora | *cinereus* | 27.0055 | -108.7850 | 366 | F |
| El Molino | Sinaloa | *sinaloa* | 24.9454 | -107.8283 | 67 | M |
| Cosalá | Sinaloa | *sinaloa* | 24.2133 | -106.8950 | 248 | M |
| Panuco | Sinaloa | *sinaloa* | 23.4167 | -105.9100 | 632 | M |
| Los Limones | Sinaloa | *sinaloa* | 23.3802 | -106.3863 | 85 | M |
| La Guásima | Sinaloa | *sinaloa* | 23.3171 | -105.9701 | 245 | M |
| Ejido Mesillas | Sinaloa | *sinaloa* | 23.1768 | -105.9900 | 223 | M |
| Cacalotan | Sinaloa | *sinaloa* | 23.0717 | -105.8033 | 61 | M |
| El Rosario | Sinaloa | *sinaloa* | 22.9770 | -105.9030 | 98 | M |
| Colorado de la Mora | Nayarit | *sinaloa* | 21.6882 | -104.6649 | 501 | M |
| Jumatán | Nayarit | *sinaloa* | 21.6445 | -105.0304 | 349 | M |
| Singayta | Nayarit | *sinaloa* | 21.5710 | -105.2337 | 26 | F |
| Mecatán | Nayarit | *sinaloa* | 21.5478 | -105.1292 | 256 | F |
| San Blas | Nayarit | *sinaloa* | 21.5238 | -105.2576 | 27 | M |
| Cerro San Juan | Nayarit | *sinaloa* | 21.5267 | -105.0433 | 870 | M |
| Cuarenteño | Nayarit | *sinaloa* | 21.4769 | -105.0018 | 1404 | F |
| Malinal Road | Nayarit | *sinaloa* | 21.3682 | -105.0245 | 783 | M |
| Tepic-Compostela | Nayarit | *sinaloa* | 21.2326 | -104.8467 | 1024 | M |
| Chacala | Nayarit | *sinaloa* | 21.1455 | -105.2248 | 151 | F |
| Puerto Vallarta | Jalisco | *sinaloa* | 20.6801 | -105.1745 | 250 | M |
| Mismaloya | Jalisco | *sinaloa* | 20.5268 | -105.2658 | 263 | F |
| Rio Horcones | Jalisco | *sinaloa* | 20.4602 | -105.2732 | 591 | F |
| Nacastillo | Jalisco | *sinaloa* | 19.6167 | -104.0208 | 1016 | F |
| Ranchitos | Jalisco | *sinaloa* | 19.6139 | -105.0208 | 190 | F |
| Santa Cruz | Jalisco | *sinaloa* | 19.6003 | -105.0453 | 113 | F |
| Fortuna | Jalisco | *sinaloa* | 19.6000 | -105.1033 | 31 | F |
| SNTE 47 | Jalisco | *sinaloa* | 19.5458 | -105.0825 | 22 | F |
| Ejido-Ari | Jalisco | *sinaloa* | 19.5392 | -105.0636 | 41 | F |
| UDG | Jalisco | *sinaloa* | 19.5264 | -105.0592 | 18 | F |
| Embrujo | Jalisco | *sinaloa* | 19.5214 | -105.0683 | 61 | F |
| UNAM-Chamela | Jalisco | *sinaloa* | 19.5103 | -105.0369 | 46 | M |
| Eje central | Jalisco | *sinaloa* | 19.5103 | -105.0369 | 46 | F |
| Limoncito | Jalisco | *sinaloa* | 19.4639 | -104.9364 | 67 | F |
| Hidalgo Viejo | Jalisco | *sinaloa* | 19.3650 | -104.8911 | 31 | F |
| Piratas | Jalisco | *sinaloa* | 19.3631 | -104.9625 | 102 | F |
| Hidalgo Nuevo | Jalisco | *sinaloa* | 19.3314 | -104.8953 | 65 | F |
| Tamarindo | Jalisco | *sinaloa* | 19.2679 | -104.7623 | 203 | F |
| Melaque | Jalisco | *sinaloa* | 19.2267 | -104.7164 | 27 | F |
| 18 km SE Colima | Colima | *sinaloa* | 19.1317 | -103.5950 | 471 | M |
| Tecomán | Colima | *sinaloa* | 18.9543 | -103.8953 | 52 | F |
| Villa Victoria | Michoacán | *sinaloa* | 18.7628 | -103.3649 | 690 | M |
| La Mira | Michoacán | *sinaloa* | 18.0951 | -102.3960 | 169 | M |
| Vallecitos de Zaragoza | Guerrero | *russeus* | 17.9500 | -101.3833 | 668 | M |
| Chilpanchingo | Guerrero | *russeus* | 17.4833 | -99.7333 | 1480 | M |
| Atoyac de Álvarez | Guerrero | *russeus* | 17.4167 | -100.1167 | 1598 | M |
| Coyuca de Benitez | Guerrero | *russeus* | 17.3333 | -99.8350 | 1258 | M |
| Agua de Obispo | Guerrero | *russeus* | 17.2667 | -99.5167 | 702 | M |
| Rio Santiago | Guerrero | *russeus* | 17.2529 | -100.3145 | 721 | F |
| Camino San Andrés | Guerrero | *russeus* | 17.2522 | -100.3427 | 798 | F |
| Atoyac-Río Santiago | Guerrero | *russeus* | 17.2506 | -100.3671 | 515 | F |
| San Miguel Reyes | Oaxaca | *russeus* | 16.9166 | -97.8899 | 769 | F |
| Santa María Tortolita | Oaxaca | *russeus* | 16.9333 | -97.9001 | 871 | F |
| San Pedro Siniyuvi | Oaxaca | *russeus* | 16.9815 | -97.8379 | 867 | F |
| Concepción del Progreso | Oaxaca | *russeus* | 17.0512 | -97.8633 | 887 | F |
| San Juan Teponaxtla | Oaxaca | *russeus* | 17.0284 | -97.8482 | 875 | F |
