# Supplementary material for: Color and morphological differentiation in the Sinaloa Wren (Thryophilus sinaloa) in the tropical dry forests of Mexico: The role of environment and geographic isolation
Source: PLoS One. 2022 Jun 23;17(6):e0269860. doi: 10.1371/journal.pone.0269860 (PMC9223310; doi:10.1371/journal.pone.0269860)
Supplement: S2 Table — Statistically significant differences between sexes within subspecies are highlighted in bold after Bonferroni corrections *** <0.001, **<0.01. ns = non-significant comparisons. (DOCX) [file pone.0269860.s006.docx]

**S2 Table. Measurements of five morphological traits in male and female Sinaloa Wren subspecies (*cinereus, sinaloa* and *russeus*).** Statistically significant differences between sexes within subspecies are highlighted in bold after Bonferroni corrections ******* <0.001, ******<0.01. ns = non-significant comparisons.

| **Including field and museum measurements** | | | | | | | | | | | | |
| --- | --- | --- | --- | --- | --- | --- | --- | --- | --- | --- | --- | --- |
|  | ***cinereus*** | |  |  | ***sinaloa*** | |  |  | ***russeus*** | |  |  |
| **Trait** | **Males**  (*n*= 13) | **Females**  (*n*= 4) | **W**  **value** | ***P*** | **Males**  (*n*= 108) | **Females**  (*n*= 30) | **W**  **value** | ***P*** | **Males**  (*n*= 34) | **Females**  (*n*= 13) | **W**  **value** | ***P*** |
| Wing length (mm) | 58.3 ± 2.0 | 54.0 ± 1.1 | 1 | ****** | 58.4 ± 1.7 | 54.0 ± 2.0 | 164 | ******* | 60.8 ± 2.0 | 55.7 ± 2.3 | 19.5 | ******* |
| Tail length (mm) | 49.1 ± 2.7 | 46.4 ± 0.7 | 9 | ns | 46.4 ± 2.3 | 41.0 ± 2.7 | 190.5 | ******* | 50.1 ± 3.9 | 46.1 ± 2.6 | 75.5 | ******* |
| Head (mm) | 37.3 ± 0.5 | 36.5 ± 0.5 | 5.5 | ns | 36.1 ± 1.2 | 34.4 ± 1.3 | 407.5 | ******* | 36.8 ± 1.0 | 35.3 ± 0.9 | 59 | ******* |
| Tarsus (mm) | 21.4 ± 0.5 | 20.3 ± 0.5 | 2.5 | ****** | 21.0 ± 1.8 | 19.8 ± 1.0 | 612.5 | ******* | 19.9 ± 1.2 | 20.1 ± 1.1 | 252.5 | ns |
| Exposed culmen (mm) | 17.1 ± 0.6 | 16.3 ± 0.5 | 8.5 | ns | 15.7 ± 1.0 | 14.8 ± 1.1 | 899.5 | ******* | 15.5 ± 1.1 | 15.1 ± 1.1 | 184.5 | ns |
| **Including only field measurements** | | | | | | | | | | | | |
|  | ***cinereus*** | |  |  | ***sinaloa*** | |  |  | ***russeus*** | |  |  |
|  | **Males**  (*n*= 13) | **Females**  (*n*= 4) | **W**  **value** | ***P*** | **Males**  (*n*= 81) | **Females**  (*n*= 14) | **W**  **value** | ***P*** | **Males**  (*n*= 26) | **Females**  (*n*= 9) | **W**  **value** | ***P*** |
| Wing length (mm) | 58.3 ± 2.0 | 54.0 ± 1.1 | 1 | ****** | 58.4 ± 1.4 | 53.9 ± 1.4 | 12 | ******* | 60.9 ± 1.7 | 55.1 ± 2.3 | 5 | ******* |
| Tail length (mm) | 49.1 ± 2.7 | 46.4 ± 0.7 | 9 | ns | 47.0 ± 1.6 | 42.4 ± 2.0 | 47 | ******* | 51.2 ± 3.3 | 46.9 ± 2.4 | 34 | ****** |
| Head (mm) | 37.3 ± 0.5 | 36.5 ± 0.5 | 5.5 | ns | 36.4 ± 0.8 | 35.2 ± 0.5 | 111 | ******* | 37.1 ± 0.8 | 35.3 ± 0.9 | 17.5 | ****** |
| Tarsus (mm) | 21.4 ± 0.5 | 20.3 ± 0.5 | 2.5 | ****** | 21.0 ± 1.3 | 19.6 ± 1.0 | 191 | ******* | 19.5 ± 1.0 | 19.7 ± 0.9 | 134.5 | ns |
| Exposed culmen (mm) | 17.1 ± 0.6 | 18.3 ± 0.5 | 8.5 | ns | 16.0 ± 0.9 | 15.6 ± 0.7 | 398 | ns | 15.7 ± 1.0 | 15.3 ± 0.8 | 97.5 | ns |
