# Supplementary material for: Color and morphological differentiation in the Sinaloa Wren (Thryophilus sinaloa) in the tropical dry forests of Mexico: The role of environment and geographic isolation
Source: PLoS One. 2022 Jun 23;17(6):e0269860. doi: 10.1371/journal.pone.0269860 (PMC9223310; doi:10.1371/journal.pone.0269860)
Supplement: S3 Table — *** Significant values < 0.001, ** <0.01, * <0.05 are highlighted in bold, ns = not significant (P > 0.05). (DOCX) [file pone.0269860.s007.docx]

**S3 Table. Mixed effects full models over phenotypical traits of *Thryophilus sinaloa*.** ******* Significant values < 0.001, ****** <0.01, ***** <0.05 are highlighted in bold, ns = not significant (*P* > 0.05).

|  | **Estimate** | **Std. Error** | **t value** |
| --- | --- | --- | --- |
| ***Morphology*** |  |  |  |
| **Wing chord** |  |  |  |
| (Intercept) | 34.20 | 10.21 | 3.34 |
| Elevation | 0.003 | 0.001 | **3.15**** |
| Evapotranspiration dry | -0.002 | 0.01 | -0.16 |
| Evapotranspiration rain | 0.04 | 0.02 | 1.56 |
| Tree cover | 0.002 | 0.01 | 0.19 |
| Annual mean temperature | 0.68 | 0.32 | **2.10*** |
| Annual precipitation | 0.0004 | 0.001 | 0.40 |
| Latitude | 0.05 | 0.11 | 0.48 |
| Sex | 4.38 | 0.31 | **13.90***** |
| **Tail length** |  |  |  |
| (Intercept) | -6.93 | 14.22 | -0.48 |
| Elevation | 0.006 | 0.001 | **4.36***** |
| Evapotranspiration dry | -0.02 | 0.02 | -1.26 |
| Evapotranspiration rain | 0.06 | 0.03 | 1.74 |
| Tree cover | -0.01 | 0.01 | -1.13 |
| Annual mean temperature | 1.41 | 0.44 | **3.15**** |
| Annual precipitation | 0.002 | 0.001 | 1.88 |
| Latitude | 0.51 | 0.15 | **3.28**** |
| Sex | 3.92 | 0.41 | **9.48***** |
| **Exposed culmen** |  |  |  |
| (Intercept) | 8.15e+00 | 5.53e+00 | 1.47 |
| Elevation | 7.16e-04 | 5.94e-04 | 1.20 |
| Evapotranspiration dry | 7.97e-03 | 9.13e-03 | 0.87 |
| Evapotranspiration rain | -1.70e-02 | 1.42e-02 | -1.19 |
| Tree cover | **1.62e-02** | **6.96e-03** | **2.33*** |
| Annual mean temperature | 1.38e-01 | 1.72e-01 | 0.80 |
| Annual precipitation | -2.25e-06 | 5.51e-04 | -0.004 |
| Latitude | 1.06e-01 | 6.22e-02 | 1.70 |
| Sex | **6.88e-01** | **1.36e-01** | **5.06***** |
| **Tarsus length** |  |  |  |
| (Intercept) | 1.17e+01 | 7.11e+00 | 1.64 |
| Elevation | 5.96e-04 | 7.68e-04 | 0.77 |
| Evapotranspiration dry | 2.03e-03 | 1.19e-02 | 0.17 |
| Evapotranspiration rain | 1.66e-02 | 1.83e-02 | 0.90 |
| Tree cover | 4.25e-03 | 9.09e-03 | 0.46 |
| Annual mean temperature | 1.68e-01 | 2.23e-01 | 0.75 |
| Annual precipitation | 1.78e-04 | 7.19e-04 | 0.24 |
| Latitude | 1.65e-01 | 8.13e-02 | **2.03*** |
| Sex | 7.89e-01 | 1.67e-01 | **4.72***** |
|  |  |  |  |
| ***Plumage brightness*** |  |  |  |
| **Head** |  |  |  |
| (Intercept) | 8.89 | 2.94 | 3.01º |
| Annual mean temperature | -0.09 | 0.97 | -0.99 |
| Annual precipitation | -0.0006 | 0.0005 | -1.23 |
| Evapotranspiration dry | -0.12 | 0.017 | -0.72 |
| Evapotranspiration rain | 0.003 | 0.009 | 0.39 |
| NDVI dry season | 0.52 | 1.53 | 0.34 |
| NDVI rainy season | -0.04 | 2.10 | -0.02 |
| **Back** |  |  |  |
| (Intercept) | 1.62 | 2.88 | **0.56*** |
| Annual mean temperature | 0.23 | 0.09 | **2.44*** |
| Annual precipitation | -0.001 | 0.0005 | **-2.32**** |
| Evapotranspiration dry | 0.05 | 0.01 | 3.04 |
| Evapotranspiration rain | -0.0009 | 0.008 | -0.10 |
| NDVI dry season | -3.66 | 1.49 | **-2.44*** |
| NDVI rainy season | 2.48 | 2.05 | 1.20 |
| **Flank** |  |  |  |
| (Intercept) | 5.10 | 8.17 | 0.62 |
| Annual mean temperature | 0.48 | 0.26 | 1.82 |
| Annual precipitation | -0.003 | 0.001 | **-2.07** |
| Evapotranspiration dry | 0.05 | 0.05 | 1.13 |
| Evapotranspiration rain | -0.03 | 0.02 | 1.11 |
| NDVI dry season | -0.18 | 4.38 | -0.04 |
| NDVI rainy season | -3.61 | 6.12 | -0.59 |
| **Tail** |  |  |  |
| (Intercept) | -6.89 | 14.09 | -0.49 |
| Annual mean temperature | 1.17 | 0.45 | **2.60**** |
| Annual precipitation | -0.001 | 0.002 | **-0.49*** |
| Evapotranspiration dry | 0.19 | 0.08 | 2.23 |
| Evapotranspiration rain | -0.06 | 0.04 | **-1.39*** |
| NDVI dry season | -18.89 | 7.77 | -2.43 |
| NDVI rainy season  ***Plumage brightness***  **Head**  (Intercept) | 10.67  9.99 | 11.09  1.82 | 0.96  5.46 |
| Annual mean UV-B  **Back** | -0.0008 | 0.0003 | **-2.23*** |
| (Intercept) | 12.28 | 2.24 | 5.46 |
| Annual mean UV-B | -0.0011 | 0.0004 | **-2.50*** |
| **Flank**  (Intercept) | 20.95 | 6.007 | 3.48 |
| Annual mean UV-B | -0.0018 | 0.0012 | -1.507 |
| **Tail** |  |  |  |
| (Intercept) | 27.34 | 11.20 | 2.44 |
| Annual mean UV-B | -0.0017 | 0.0022 | -0.765 |
