# Supplementary material for: Color and morphological differentiation in the Sinaloa Wren (Thryophilus sinaloa) in the tropical dry forests of Mexico: The role of environment and geographic isolation
Source: PLoS One. 2022 Jun 23;17(6):e0269860. doi: 10.1371/journal.pone.0269860 (PMC9223310; doi:10.1371/journal.pone.0269860)
Supplement: S4 Table — Akaike Information Criterion (AIC) and F statistics were included. P-values representing significant differences are shown in bold. (DOCX) [file pone.0269860.s008.docx]

**S4 Table.** **Partial redundancy analysis (RDA) model selection showing environmental variables that best explained phenotypical variation (morphology and color from the avian perspective).** Akaike Information Criterion (AIC) and *F* statistics were included. *P-values* representing significant differences are shown in bold.

| ***Morphological variation*** | **AIC** | ***F*** | ***P-value*** |
| --- | --- | --- | --- |
| Precipitation of coldest quarter (Bio19) | 305.95 | 11.2856 | **0.005** |
| Evapotranspiration rainy season | 305.31 | 10.6376 | **0.005** |
| Precipitation of the driest month (Bio14) | 299.90 | 5.2578 | **0.005** |
| Elevation | 299.99 | 5.3422 | **0.010** |
| Precipitation Seasonality (Bio15) | 299.24 | 4.6076 | **0.015** |
| NDVI rainy season | 298.58 | 3.9683 | **0.015** |
| Precipitation of warmest month (Bio18) | 298.22 | 3.6119 | **0.025** |
| Mean temperature of driest quarter (Bio9) | 297.33 | 2.7509 | **0.035** |
| Precipitation of wettest month (Bio13) | 296.34 | 2.0372 | 0.075 |
| Evapotranspiration dry season | 296.39 | 1.9900 | 0.100 |
| Tree cover | 296.60 | 1.7865 | 0.130 |
| Mean diurnal range (Bio2) | 297.45 | 0.9728 | 0.340 |
| NDVI dry season | 298.01 | 0.4349 | 0.740 |
| ***Plumage color variation from avian perspective*** | | | |
| Precipitation of coldest quarter (Bio19) | 240.33 | 15.4194 | **0.005** |
| Mean diurnal range (Bio2) | 230.34 | 4.8320 | **0.005** |
| Precipitation of the driest month (Bio14) | 227.64 | 2.1515 | **0.020** |
| NDVI dry season | 227.54 | 1.8088 | 0.075 |
| Precipitation of warmest month (Bio18) | 228.15 | 1.2245 | 0.180 |
| Precipitation Seasonality (Bio15) | 228.12 | 1.2540 | 0.230 |
| Evapotranspiration dry season | 228.30 | 1.0775 | 0.350 |
| Mean temperature of driest quarter (Bio9) | 228.50 | 0.8864 | 0.500 |
| Elevation | 228.56 | 0.8312 | 0.520 |
| Precipitation of wettest month (Bio13) | 228.46 | 0.9244 | 0.545 |
| Evapotranspiration rainy season | 228.80 | 0.5996 | 0.675 |
| Tree cover | 228.64 | 0.7509 | 0.715 |
| NDVI rainy season | 229.06 | 0.3460 | 0.990 |
