# Supplementary material for: Color and morphological differentiation in the Sinaloa Wren (Thryophilus sinaloa) in the tropical dry forests of Mexico: The role of environment and geographic isolation
Source: PLoS One. 2022 Jun 23;17(6):e0269860. doi: 10.1371/journal.pone.0269860 (PMC9223310; doi:10.1371/journal.pone.0269860)
Supplement: S5 Table — Environmental variables explaining phenotypical variation (morphology and color from the avian perspective). (DOCX) [file pone.0269860.s009.docx]

**S5 Table. Loadings of environmental variables into the first two axes of pRDA1**. Environmental variables explaining phenotypical variation (morphology and color from the avian perspective).

| ***Morphological variation*** | | | |
| --- | --- | --- | --- |
|  | ***df*** | **Axis 1** | **Axis 2** |
| Precipitation of coldest quarter (Bio19) | 2 | 0.09532 | 0.04142 |
| Evapotranspiration rainy season | 2 | -0.59619 | 0.09374 |
| Precipitation of driest month (Bio14) | 2 | 0.20073 | -0.37723 |
| Elevation | 2 | -0.06326 | 0.24081 |
| NDVI rainy season | 2 | 0.24189 | 0.02353 |
| Precipitation seasonality (Bio15) | 2 | -0.09929 | 0.47749 |
| Precipitation of warmest quarter (Bio18) | 2 | -0.34864 | 0.32448 |
| Mean temperature of driest quarter (Bio9) | 2 | 0.04822 | 0.05962 |
| ***Plumage color variation from avian perspective*** | | | |
| Precipitation of coldest quarter (Bio19) | 2 | 0.08591 | 0.3667 |
| Mean diurnal range (Bio2) | 2 | -0.56809 | -0.0464 |
| Precipitation of driest month (Bio14) | 2 | 0.26483 | 0.3007 |
