# Supplementary material for: Color and morphological differentiation in the Sinaloa Wren (Thryophilus sinaloa) in the tropical dry forests of Mexico: The role of environment and geographic isolation
Source: PLoS One. 2022 Jun 23;17(6):e0269860. doi: 10.1371/journal.pone.0269860 (PMC9223310; doi:10.1371/journal.pone.0269860)
Supplement: S6 Table — Summary of factor loadings and proportion of variance of the climatic indexes for first principal component in a PCA. (DOCX) [file pone.0269860.s010.docx]

**S6 Table. Summary of factor loadings and proportion of variance of the climatic indexes for the first principal component in a PCA.** Summary of factor loadings and proportion of variance of the climatic indexes for first principal component in a PCA.

| **Index** | **Proportion of variance** | **PC1** | |
| --- | --- | --- | --- |
| Temperature variation | 77.28% | BIO1 | -0.383 |
|  |  | BIO5 | -0.201 |
|  |  | BIO6 | -0.473 |
|  |  | BIO8 | -0.396 |
|  |  | BIO9 | -0.361 |
|  |  | BIO10 | -0.379 |
|  |  | BIO11 | -0.397 |
| Temperature range/  seasonality | 99.88% | BIO2 | 0.009 |
|  |  | BIO3 | -0.005 |
|  |  | BIO4 | 0.999 |
|  |  | BIO7 | 0.031 |
| Variation of precipitation in humid season | 89.19% | BIO12 | 0.658 |
|  |  | BIO13 | 0.186 |
|  |  | BIO16 | 0.532 |
|  |  | BIO18 | 0.499 |
| Variation of precipitation in dry season | 90.02% | BIO14 | -0.044 |
|  |  | BIO15 | 0.133 |
|  |  | BIO17 | -0.215 |
|  |  | BIO19 | -0.966 |
